# KDM2A integrates DNA and histone modification signals through a CXXC/PHD module and direct interaction with HP1

**DOI:** 10.1093/nar/gkw979

**Published:** 2016-10-24

**Authors:** Julie Borgel, Marek Tyl, Karin Schiller, Zsofia Pusztai, Christopher M. Dooley, Wen Deng, Carol Wooding, Richard J. White, Tobias Warnecke, Heinrich Leonhardt, Elisabeth M. Busch-Nentwich, Till Bartke

**Affiliations:** 1 MRC Clinical Sciences Centre (CSC), Du Cane Road, London W12 0NN, UK; 2 Institute of Clinical Sciences (ICS), Faculty of Medicine, Imperial College London, Du Cane Road, London W12 0NN, UK; 3 Wellcome Trust Sanger Institute, Wellcome Trust Genome Campus, Hinxton CB10 1SA, UK; 4 Department of Biology II, Center for Integrated Protein Science Munich, Ludwig Maximilians University (LMU Munich), 82152 Planegg-Martinsried, Germany

## Abstract

Functional genomic elements are marked by characteristic DNA and histone modification signatures. How combinatorial chromatin modification states are recognized by epigenetic reader proteins and how this is linked to their biological function is largely unknown. Here we provide a detailed molecular analysis of chromatin recognition by the lysine demethylase KDM2A. Using biochemical approaches we identify a nucleosome interaction module within KDM2A consisting of a CXXC type zinc finger, a PHD domain and a newly identified Heterochromatin Protein 1 (HP1) interaction motif that mediates direct binding between KDM2A and HP1. This nucleosome interaction module enables KDM2A to decode nucleosomal H3K9me3 modification in addition to CpG methylation signals. The multivalent engagement with DNA and HP1 results in a nucleosome binding circuit in which KDM2A can be recruited to H3K9me3-modified chromatin through HP1, and HP1 can be recruited to unmodified chromatin by KDM2A. A KDM2A mutant deficient in HP1-binding is inactive in an *in vivo* overexpression assay in zebrafish embryos demonstrating that the HP1 interaction is essential for KDM2A function. Our results reveal a complex regulation of chromatin binding for both KDM2A and HP1 that is modulated by DNA- and H3K9-methylation, and suggest a direct role for KDM2A in chromatin silencing.

## INTRODUCTION

The genetic material of eukaryotic cells is stored in the nucleus in the form of chromatin, a nucleoprotein complex composed of DNA and histone proteins. DNA and histones both carry chemical modifications creating distinct combinatorial signatures that encode epigenetic information and recruit effector proteins regulating chromatin function (1). These effector proteins must be able to integrate signals from both DNA and histone modifications in order to translate this information into physiological responses. How chromatin modification states are interpreted by epigenetic readers and how this is linked to biological processes is largely unknown.

A factor that has the potential to decode different nucleosomal modification signatures is the histone H3K36-demethylase KDM2A, the first jumonji C (JmjC) histone demethylase to be identified (2). KDM2A is a multi-domain protein consisting of the JmjC demethylase domain, a CXXC-type zinc finger (ZnF), a PHD domain, an F-box and several C-terminal leucine-rich repeats (LRR). In addition to the full-length protein KDM2A is expressed in several shorter isoforms, the most prominent of which—KDM2A_SF_ (for short form)—spans from the CXXC-ZnF to the C-terminus and lacks the catalytic JmjC domain (3–5). KDM2A was shown to recognize nucleosomal linker DNA containing unmethylated CpG-dinucleotides via its CXXC-ZnF. This interaction is blocked by DNA methylation (3,6,7). KDM2A was also found to interact with heterochromatin protein 1 (HP1) proteins (8) and HP1 was shown to recruit KDM2A to H3K9me3-modified nucleosomes (3). KDM2A, therefore, processes information from both DNA and histone modification marks and has the potential to distinguish between different chromatin states. KDM2A was shown to have a function at CpG island promoters (7) and in heterochromatin maintenance (8), and to regulate rRNA gene transcription during starvation (5,9). There is increasing evidence that KDM2A is involved in cancer formation (10–16) but the underlying molecular mechanisms remain unclear.

Here, we present a detailed molecular analysis of how KDM2A is recruited to chromatin and how it distinguishes between different nucleosomal modification states. Using *in vitro* reconstituted modified nucleosomes and nucleosome interaction assays we have mapped an extended chromatin recognition module in KDM2A comprising the CXXC-ZnF, the PHD domain and a newly identified variant HP1-binding motif. We demonstrate that this HP1-binding motif mediates a direct interaction between KDM2A and the HP1 chromo shadow domain. The resulting multivalent interaction enables a complex binding circuit on chromatin involving both factors which is modulated by DNA- and H3K9-methylation and which allows KDM2A and HP1 to be targeted to chromatin in a DNA or H3K9me3-independent manner, respectively. The HP1-motif is enriched for cancer mutations and we find that an HP1 binding-deficient KDM2A mutant is inactive in an *in vivo* overexpression assay in zebrafish embryos underscoring the importance of HP1 binding for KDM2A function. Our results reveal a complex regulation of chromatin binding for both KDM2A and HP1, and suggest a direct role for KDM2A in chromatin silencing.

## MATERIALS AND METHODS

### Tissue culture and immunofluorescence

293T, BHK and HeLa cells were grown in DMEM medium supplemented with 10% FBS. 293T cells were transfected using calcium phosphate or polyethylenimine (PEI) protocols, and BHK and HeLa cells were transfected using PEI. Mouse ES cells (mixed 129/SV-C57B1/6J WT cells and *Suv39h* dn derivative (17)) were grown on 0.2% (w/v) gelatin-coated dishes in DMEM medium containing high glucose, 15% (v/v) fetal calf serum, 100 U/ml penicillin, 100 μg/ml streptomycin, 2 mM l-glutamine, 0.1 mM β-mercaptoethanol, 1× non-essential amino acids, and LIF. Transfections with FLAG-GFP-KDM2A expression constructs were performed using Fugene HD transfection reagent (Promega) according to the manufacturer's recommendations for the transfection of mouse stem cells. For immunofluorescence (IF) analysis, cells were collected by trypsinization and immobilized on slides by cytospin. The cells were then washed in PBS and fixed in PBS containing 0.3% Triton X-100 and 3.7% formaldehyde for 10 min. After two washes in PBS, cells were blocked in blocking buffer (PBS containing 10% milk; 3% BSA; 0.2% Tween and 0.2% NP-40) for 20 min at RT. The cells were then incubated for 1 h with 0.5 μg/ml of each primary antibody (rabbit anti-GFP for GFP-KDM2A detection and mouse anti-HP1α) and for 30 min with DAPI and the secondary antibodies where required in the dark. The cells were mounted in ProLong Gold DAPI-free mounting medium (LifeTechnologies). Images were acquired with a TCS SP5 laser scanning confocal microscope (Leica Microsystems GmbH) and processed using ImageJ. Twenty to fifty cells from one to three experiments for each construct and condition were scored based on co-localization of the GFP-KDM2A signal with DAPI dense foci.

### Extract preparation and co-immunoprecipitations

293T whole cell extracts were prepared ∼36 h after transfection by rotating the cells in extraction buffer (20 mM Hepes pH 7.5; 300 mM NaCl; 1 mM EDTA; 20% glycerol; 0.5% NP40; 1 mM DTT and complete protease inhibitors [Roche]) for 1 h at 4°C and removing cell debris by centrifugation. HeLa S3 cells were grown in suspension in RPMI 1640 medium containing 10% FBS. Cells were harvested at a density of 0.5–0.8×10^6^ cells/ml and nuclear extracts were essentially prepared as described (18). All extracts were snap frozen and stored in aliquots at −80°C. For co-immunoprecipitation of endogenous KDM2A and HP1 proteins (Figure 2A) nuclear extracts were prepared from HeLa cells using a modified protocol combining the soluble and chromatin-bound fractions. Briefly, nuclear pellets were resuspended in five pellet volumes of low salt solubilization buffer (20 mM Hepes pH7.5; 150 mM NaCl; 1.5 mM MgCl_2_; 0.2 mM EDTA; 25% Glycerol; 0.1% NP40; 0.5 mM DTT; 0.5 mM PMSF and complete protease inhibitors) supplemented with 200 U/ml Benzonase (Sigma) and incubated at 37°C for 15 min. After a 10 s sonication step on ice insoluble chromatin was pelleted and the supernatant kept on ice (Fraction I). The pellets were resuspended in high salt solubilization buffer (20 mM Hepes pH7.5; 500 mM NaCl; 1.5 mM MgCl_2_; 0.2 mM EDTA; 25% glycerol; 0.1% NP40; 0.5 mM DTT; 0.5 mM PMSF and complete protease inhibitors) and rotated at 4°C for 1 h. After three 10 s sonication steps debris was pelleted by centrifugation and the resulting supernatant (Fraction II) combined with Fraction I. Precipitates were removed by centrifugation and the nuclear extracts were snap frozen and stored in aliquots at −80°C. Immunoprecipitations were carried out with 1 mg of nuclear extract in a total volume of 1 ml in the presence of 50 μg of ethidium bromide. Extracts were pre-cleared with Dynabeads Protein G (Invitrogen) for 1 h at 4°C and then incubated with anti-GFP or anti-KDM2A antibodies for 3 h at 4°C. Antibody-bound proteins were captured with Dynabeads Protein G for 2 h at 4°C. After five washes with wash buffer (20 mM Hepes pH7.5; 150 mM NaCl; 0.2 mM EDTA; 20% glycerol; 0.1% NP40 and complete protease inhibitors) bound proteins were eluted in sample buffer and analyzed by SDS-PAGE and immunoblot. For co-immunoprecipitation of FLAG-GFP-tagged KDM2A and FLAG-tagged HP1α (Figure 2B and C) transiently transfected HeLa cells were lysed in high salt extraction buffer (20 mM Hepes pH 7.5; 375 mM NaCl; 1 mM EDTA; 20% glycerol; 0.5% NP40; 1 mM PMSF and complete protease inhibitors) for 2 h at 4°C. Cell debris was removed by centrifugation and the extracts were then diluted 2.5-fold with 20 mM Hepes pH7.5; 1 mM EDTA; 20% glycerol; containing 0.5 mM PMSF and complete protease inhibitors to adjust the NaCl concentration to 150 mM. The diluted extracts were rotated for 2 h at 4°C with 20 μl of magnetic GFP-Trap beads (Chromotek) in order to immunoprecipitate GFP-tagged proteins. After four washes with wash buffer bound proteins were eluted in sample buffer and analyzed by SDS-PAGE followed by immunoblot.

### *In vitro* KDM2A/HP1 interaction assays

For analysis of KDM2A_543-811_/HP1 complexes by size exclusion chromatography 50 μg of each protein were mixed in a total volume of 50 μl and incubated for 15 min at 4°C. The complete binding reaction was separated on a Superdex 200 Increase 3.2/300 column in gel filtration buffer (50 mM Tris pH7.5, 100 mM NaCl, 5 mM β-mercaptoethanol) collecting 50 μl fractions. Ten microliters of each fraction were analyzed by SDS-PAGE followed by Coomassie staining. For analysis of the interaction between full-length KDM2A cancer mutants and recombinant HP1α FLAG-GFP-tagged KDM2A variants were transiently expressed in 293T cells and whole cell extracts were prepared in extraction buffer (20 mM Hepes pH7.5; 300 mM NaCl; 1 mM EDTA; 20% glycerol; 0.5% NP40; 1 mM PMSF and complete protease inhibitors). For each mutant, three separate extracts were prepared and pooled to even out differences in expression levels. Relative expression levels of KDM2A cancer mutants in the pooled extracts were determined by quantitative immunoblot against the FLAG tag using a LAS4000 mini chemiluminescence imaging system and ImageQuant software (GE Healthcare). KDM2A expressing extracts were then adjusted with mock-transfected extracts to achieve similar expression levels for all mutants. For pull-downs 0.5 mg (mutant screen) or 0.2 mg (WT versus K793N) of extract were rotated with anti-FLAG M2 agarose beads (Sigma) in extraction buffer for 3 h at 4°C to capture FLAG-GFP-tagged KDM2A. The beads were washed twice with extraction buffer followed by one wash in binding buffer (20 mM HEPES pH 7.9, 150 mM NaCl, 0.2 mM EDTA, 20% glycerol, 0.1% NP40 and complete protease inhibitors) and immobilized KDM2A was then rotated in 200 μl binding buffer with recombinant purified HP1α as indicated for 2 h at 4°C. After three washes in binding buffer bound proteins were eluted in sample buffer and analyzed by SDS-PAGE and immunoblot. Chemiluminescence signals were detected using a LAS4000 mini imaging system and quantified using ImageQuant software (GE Healthcare).

### Reconstitution of modified nucleosomes and nucleosome pull-downs

**Figure 1.**
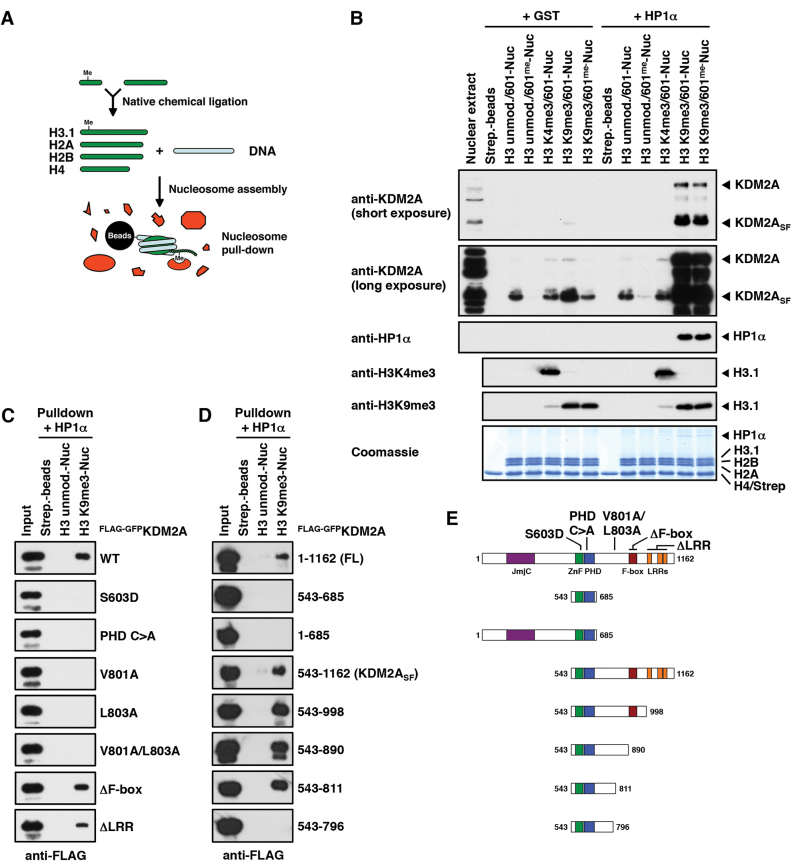
Mapping of nucleosome binding sites within KDM2A. (**A**) Schematic representation of the assembly of modified nucleosomes and nucleosome pull-downs. Modified histone H3.1 was prepared by native chemical ligation and assembled into nucleosomes together with purified core histones H2A, H2B and H4 and nucleosomal 601-DNA. Nucleosomes were immobilized on streptavidin beads via the biotinylated DNA and incubated with protein extracts or recombinant proteins to identify modification-binding factors. (**B**) Recruitment of KDM2A to H3K9me3-modified nucleosomes is stimulated by HP1 and counteracted by CpG-methylation. HeLa S3 nuclear extracts were incubated with immobilized modified nucleosomes as indicated. Binding reactions were supplemented with recombinant purified HP1α or GST as a control. 4% of the nuclear extract input and 20% of the pull-downs were separated by SDS-PAGE and nucleosome-bound KDM2A and HP1α were detected by immunoblot. Equal loading was confirmed by Coomassie stain and modification of histone H3 was verified by immunoblot against H3 tri-methyl lysine marks. Both, the full length and the short isoform of KDM2A (KDM2A_SF_) show increased binding to H3K9me3-modified nucleosomes, which is strongly stimulated by addition of HP1α and reduced by CpG-methylation. (**C**) Mapping of nucleosome binding domains in KDM2A. Unmodified or H3K9me3-modified nucleosomes were immobilized on streptavidin beads and incubated with 293T whole cell extracts overexpressing wild-type (WT) FLAG-GFP-tagged KDM2A or point/deletion mutants as indicated. All binding reactions were supplemented with recombinant purified HP1α. Forty percent of each input and pull-down were separated by SDS-PAGE and binding of KDM2A was detected by immunoblot against the FLAG tag. The S603D point mutation disrupts binding of the CXXC-ZnF to CpG dinucleotides (37) and the PHD C>A mutant contains C620A and C623A point mutations disrupting the structural integrity of the PHD domain. In the ΔF-box mutant amino acids 893 to 933 and in the ΔLRR mutant amino acids 1000 to 1118 are deleted. Loading controls can be found in Supplementary Figure S2A. (**D**) Mapping of a nucleosome interaction module within KDM2A. Nucleosome binding reactions were carried out as above using FLAG-GFP-tagged full length (FL) KDM2A or N- or C-terminal deletion mutants including amino acids as indicated. Loading controls can be found in Supplementary Figure S2B. (**E**) Schematic representation of the domain structure of KDM2A and the point and deletion mutants used in Figure 1C and D.

Modified human histone H3.1 proteins were generated by native chemical ligation and assembled into nucleosomes together with purified human H2A, H2B and H4 and biotinylated 601-DNA as described (3). CpG-methylated 601-DNA was generated using M.SssI CpG methyltransferase (NEB). For nucleosome pull-downs from HeLa S3 nuclear extracts (Figure 1B) nucleosomes corresponding to 15 μg of octamer were immobilized on 80 μl Dynabeads MyOne Streptavidin T1 (Invitrogen) and incubated with 0.5 mg HeLaS3 nuclear extract in 1 ml of binding buffer (20 mM HEPES pH7.9, 150 mM NaCl, 0.2 mM EDTA, 20% glycerol, 0.1% NP40, 1 mM DTT and complete protease inhibitors) together with 6 μg of recombinant HP1α or GST for 4 h at 4°C. After three washes in binding buffer, bound proteins were eluted in sample buffer and analyzed by SDS-PAGE followed by immunoblot or Coomassie staining. Pull-downs from overexpressing 293T cell extracts were performed with immobilized nucleosomes corresponding to 2.5 μg of octamer and 1 μg of recombinant HP1α (Figure 1C, D and Supplementary Figure S2A-C) or 5 μg of octamer and 2 μg of recombinant HP1α (Supplementary Figure S1) in a total volume of 0.5 ml of binding buffer containing 10–20 μl of 293T whole cell extract. The volume of 293T whole cell extract was adjusted with mock-transfected extracts according to the expression levels of individual KDM2A mutants/deletions. Nucleosome pull-downs with purified proteins were carried out in 1 ml of binding buffer as indicated in the figure legends. For Figure 3A and B, Supplementary Figure S4A and S4B nucleosomes corresponding to 2.5 μg of histone octamer were immobilized on streptavidin beads and incubated with 2 μg of WT or mutant full length FLAG-GFP-tagged KDM2A and 2 μg of HP1α. For Figure 4A and B nucleosomes corresponding to 2.5 μg of histone octamer were immobilized on streptavidin beads and incubated with 2.5 μg of WT or mutant HP1α and/or 2.5 μg of KDM2A_543-811_. For Supplementary Figure S5 nucleosomes corresponding to 4 μg of histone octamer (∼40 pmol) were immobilized on streptavidin beads and incubated with increasing amounts of HP1α (0.05 μg; 0.5 μg; 5 μg; 40 μg) or KDM2A_543-811_ (0.12 μg; 1.2 μg; 12 μg; 40 μg) in the presence of either 23 μg of KDM2A_543-811_ (∼0.8 nmol; lanes 4–8 and 14–18) or 8 μg of HP1α (∼0.4 nmol; lanes 24–28 and 34–38). After three washes, bound proteins were eluted in sample buffer and analyzed by SDS-PAGE followed by immunoblot or Coomassie staining.

### Fluorescent three hybrid (F3H) assay

BHK cells with an integrated *lac* operator array (19) were seeded on coverslips in six-well plates and triple-transfected with the corresponding fluorescent protein fusion plasmids and a plasmid expressing LacI-fused GFP binder protein (pGBP-LacI) (20). The transfected cells were fixed with 3.7% formaldehyde, stained with DAPI, and mounted on slides in Vectashield medium (Vector Laboratories). Images of the cells were acquired with a TCS SP5 laser scanning confocal microscope (Leica Microsystems GmbH) using a 63× objective. 405, 488 and 561 nm lasers were used to excite the DAPI, GFP and mRuby2, respectively. Signals were recorded with a pixel size of 80 nm by PMT detectors. For quantitative assays, a spinning disc confocal microscope UltraView VOX (PerkinElmer Inc.) was used. For each imaging field, Z stack images with a 1 μm step size were acquired for a total depth of 6 μm and then projected according to the maximum intensity. The projection was analyzed with imageJ. To quantify the interaction between the two fluorescent fusion proteins, the fluorescence intensity ratio between the mRuby2 and GFP at the *lac*O spot were calculated. In brief, the spot area was segmented according to the GFP signal, and the average fluorescence intensity of this area in both GFP and mRuby2 channels was measured (*I*^spot GFP^ and *I*^spot mRuby2^) and the average intensity of the whole nucleus (*I*^nucleus GFP^ and *I*^nucleus mRuby2^) was subtracted to eliminate the impact of different expression levels between the channels and cells. The ratio of fluorescence intensity at the spot between mRuby2 and GFP were then calculated by (*I*^spot mRuby2^ − *I*^nucleus mRuby2^)/ (*I*^spot GFP^ − I^nucleus GFP^), as an indication of interaction between the two proteins.

### Overexpression of human KDM2A in zebrafish embryos

Zebrafish embryos of the TLF (Tüpfel long fin) strain were injected at the one cell stage with 3.6 nl of capped polyadenylated mRNA (mMESSAGE mMACHINE T7, Ambion) generated from human FLAG-GFP-tagged KDM2A constructs (see Supplementary Methods) at a concentration of 62.5 ng/μl combined with 0.05% Phenol Red (Sigma). Embryos were raised at 28.5°C for 24 h and then characterized based on their morphology. Dead embryos and embryos with abnormal phenotypes were classified into the abnormal category.

### Statistical analysis of overexpression experiments

Overexpression experiments were analysed using Fisher's Exact Test. For the V801A/L803A vs WT experiment a 2 × 3 contingency table was first used to assess if there was any difference between any of the groups and then each pair-wise comparison was performed. The *P*-values were then adjusted for multiple testing using Bonferroni correction.

### Sequence alignments, SNPs and cancer SNVs

KDM2A orthologues from species representing different levels of phylogenetic depth (mouse, chicken, green anole, *Xenopus tropicalis*, zebrafish) along with select paralogues (human KDM2B, zebrafish Kdm2ab) and more distant members of the same gene family (human FBXL19, *Ciona intestinalis* ZF(CXXC)-1) were identified using Treefam (TF106480; www.treefam.org) (21). Protein sequences were downloaded from Ensembl v75 (22) and aligned using Muscle v3.8.31 (23). The alignment is provided as Supplementary File 1 in a CLC Main Workbench 6 format (CLC Bio). Missense SNPs in KDM2A (ENSG00000173120) segregating in the human population were obtained from Ensembl v81 (22). Missense cancer SNVs were compiled from Ensembl which includes COSMIC v71 (24) and cBioPortal/TCGA (25,26), with redundant entries removed after cross-checking tumor sample IDs. For TCGA data, curated rather than automatically generated MAF files were used where available. Three variants were present as both a cancer SNV and a SNP. These SNPs/SNVs were excluded from Figure 7A. A complete list of SNPs/SNVs in KDM2A is provided as Supplementary Table S1.

## RESULTS

### KDM2A contains an extended nucleosome recognition module

We previously identified KDM2A in a SILAC nucleosome affinity purification screen for nucleosome-binding proteins with differential responses to DNA and histone-methylation marks (3). We found KDM2A to be enriched on nucleosomes bearing the heterochromatic H3K9me3-modification and to be prevented from binding by DNA methylation. We found that the binding to H3K9me3-modified nucleosomes was mediated by HP1 proteins (3), principle components of heterochromatin known to bind to the H3K9me3-mark and to recruit other chromatin silencing factors (27,28). In an effort to further understand how KDM2A is targeted to chromatin we set out to dissect the binding mechanisms of KDM2A to modified nucleosomes. We first assembled nucleosomes combining histone H3 tri-methylated at lysine 4 or 9 generated by native chemical ligation (29) and DNA methylated at CpG-di-nucleotides and incubated these with HeLaS3 nuclear extracts (Figure 1A). As observed in our previous experiments KDM2A present in the extracts displayed basal binding to unmodified nucleosomes that was increased on H3K9me3-modified nucleosomes and inhibited by CpG methylation (Figure 1B). KDM2A was reported to interact with all HP1 isoforms (8). Indeed, addition of purified recombinant HP1α resulted in strong recruitment of KDM2A to H3K9me3-modified nucleosomes (Figure 1B). Overexpressed FLAG-GFP-tagged KDM2A in 293T whole cell extracts recapitulated the stimulation of binding by HP1α although enrichment on H3K9me3-modified nucleosomes without HP1 addition was less pronounced, most likely due to the excess of overexpressed KDM2A over endogenous HP1 proteins (Supplementary Figure S1).

In order to identify the binding sites within KDM2A required for the nucleosome interaction we incubated unmodified and H3K9me3-modified nucleosomes with 293T extracts expressing mutant forms of KDM2A in the presence of purified HP1α. Systematic deletion and point mutants of individual domains showed that both the ZnF and the PHD domain were necessary for efficient nucleosome recognition (Figure 1C and Supplementary Figure S2A). However, a fragment encompassing only the ZnF and PHD domains did not show strong interaction (Figure 1D and Supplementary Figure S2B) indicating the requirement for an additional binding determinant in KDM2A, possibly an HP1-interaction motif. Since mutation of two apparent PxVxV HP1-binding motifs (30,31) centred at position V530 and V835 had no effect we set out to identify this site by systematic large-scale deletions (Figure 1D, E and Supplementary Figure S2B). A mutant lacking the complete C-terminal sequence following the PHD domain lost efficient binding while the previously described isoform KDM2A_SF_ (3–5) displayed binding properties similar to the full-length (FL) protein, indicating the presence of an additional binding site in the C-terminal region of KDM2A. Further C-terminal deletions of the KDM2A_SF_ variant revealed a minimal nucleosome-interacting fragment ranging from amino acids 543–811. Removal of another 15 amino acids from the C-terminus of this fragment resulted in loss of efficient nucleosome binding. Analysis of the sequence of these amino acids indicated the presence of a variant LxVxL HP1-interaction motif (Figure 7A) found in a minority of HP1-interacting proteins (30). An alanine point mutant scan of the motif (Supplementary Figure S2C) identified valine 801 and leucine 803 to be critical amino acids for efficient nucleosome binding of KDM2A (Figure 1C and Supplementary Figure S2A). Thus, KDM2A harbours an extended ‘nucleosome recognition module’ ranging from amino acids 543–811, which contains the CXXC-ZnF, the PHD domain and a variant HP1 interaction motif, all of which are required for efficient binding to H3K9me3-modified nucleosomes in the presence of HP1.

### KDM2A directly interacts with the HP1 chromo shadow domain via its LxVxL motif

**Figure 2.**
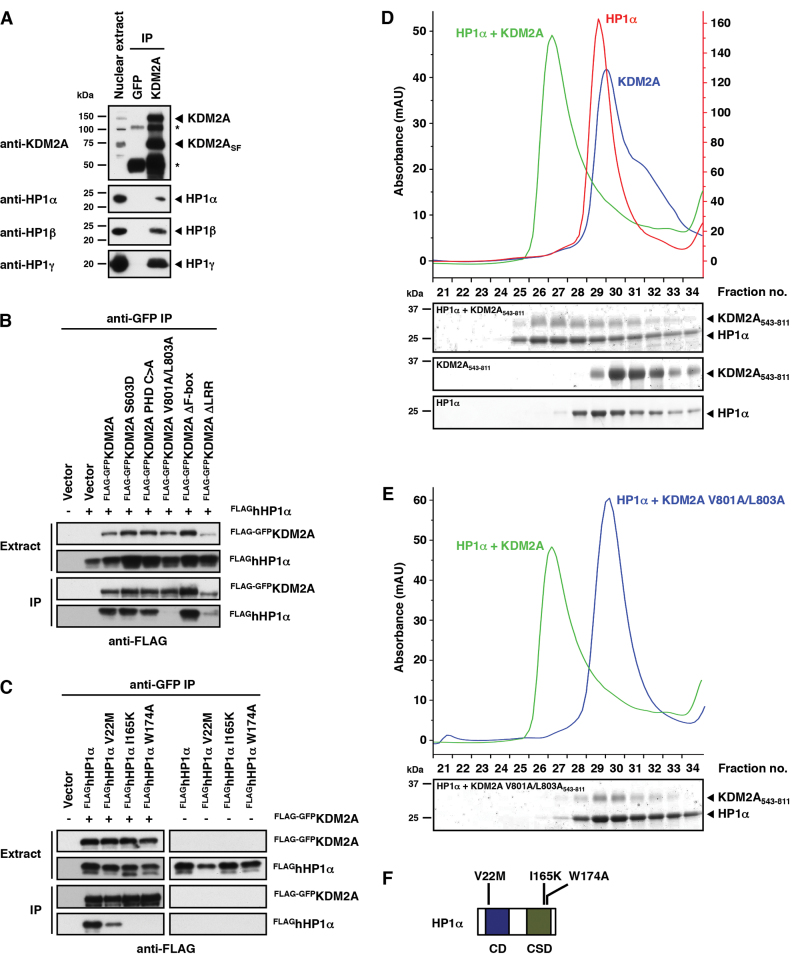
KDM2A binds to the HP1 chromo shadow domain via a variant HP1 interaction motif. (**A**) KDM2A interacts with all HP1 isoforms. Endogenous KDM2A was immunoprecipitated from HeLa nuclear extracts consisting of the soluble and chromatin-bound nuclear fractions. One percent of the nuclear extract input and 30% of the IPs were separated by SDS-PAGE and co-immunoprecipitating endogenous HP1 proteins were detected by immunoblot against HP1 α, β and γ, respectively. The KDM2A antibody immunopreciptates both the full length and the short form of KDM2A (KDM2A_SF_). The asterisks denote bands derived from the antibodies used for IP. (**B**) KDM2A interacts with HP1α via its LxVxL HP1-binding motif. FLAG-GFP-tagged WT KDM2A or point/deletion mutants were co-expressed with FLAG-tagged HP1α in HeLa cells. KDM2A was immunoprecipitated from whole cell extracts using GFP-trap beads and bound KDM2A and HP1α were detected by immunoblot against the FLAG tag. (**C**) HP1α interacts with KDM2A via the chromo shadow domain. FLAG-GFP-tagged WT KDM2A was co-expressed with FLAG-tagged WT or mutant HP1α in HeLa cells as indicated. KDM2A was immunoprecipitated from whole cell extracts using GFP-trap beads and bound KDM2A and HP1α were detected by immunoblot against the FLAG tag. (**D**) The interaction between KDM2A and HP1 is direct. Recombinant purified HP1α and the purified KDM2A nucleosome recognition module (KDM2A_543-811_) were mixed and then separated by size exclusion chromatography. Formation of a complex is indicated by a shifted co-migration of both proteins in higher molecular weight fractions. The figure shows chromatograms of the complex and the individual proteins and Coomassie-stained gels of individual fractions separated by SDS-PAGE. (**E**) Size exclusion chromatography shows that HP1α fails to form a complex with a KDM2A nucleosome recognition module harbouring the V801A/L803A mutation and confirms that the KDM2A/HP1 interaction is mediated by the LxVxL-interaction motif. The chromatogram of the complex formed between the WT proteins is shown for comparison (green trace). (**F**) Schematic representation of the domain structure of HP1α indicating the positions of inactivating point mutations in the chromo domain (CD) and chromo shadow domain (CSD).

To verify a potential direct interaction between KDM2A and HP1 we first immunoprecipitated endogenous KDM2A from HeLa nuclear extracts prepared by combining both soluble and chromatin-bound nuclear fractions. Western blots against endogenous HP1 isoforms α, β, and γ demonstrated that KDM2A could co-immunoprecipitate all three HP1 isoforms (Figure 2A).

We next investigated whether the LxVxL motif is involved in this interaction. To this end we co-expressed various point and deletion mutants of FLAG-GFP-tagged KDM2A (see Figure 1E) and FLAG-tagged HP1α (Figure 2F) in HeLa cells and immuno-precipitated KDM2A from whole cell extracts via the GFP tag. Bound HP1α was detected by western blot against the FLAG tag (Figure 2B and C). These experiments demonstrate that the V801A/L803A mutation in the LxVxL motif disrupts the KDM2A/HP1α interaction (Figure 2B). A V22M point mutant in the chromo domain (CD) of HP1α that interferes with the interaction of HP1 with the histone H3 N-terminal tail (32) retained KDM2A binding, whereas I165K and W174A point mutations in the chromo shadow domain (CSD) that interfere with the binding of HP1 to factors containing P/LxVxV/L/M interaction motifs (30,33) disrupted the binding to KDM2A (Figure 2C).

These findings were corroborated in a direct interaction assay in which we incubated recombinantly purified HP1α with a purified version of the KDM2A nucleosome recognition module ranging from the ZnF to the HP1 interaction motif (amino acids 543–811). Separation of the proteins by size exclusion chromatography showed the formation of a KDM2A/HP1α complex demonstrating a direct interaction between KDM2A and HP1α (Figure 2D). Further size exclusion chromatography experiments using the KDM2A V801A/L803A mutant, the HP1α I165K and W174A mutants, and purified HP1β and HP1γ (Figure 2E and Supplementary Figure S3A-D) confirm direct binding between KDM2A and all HP1 isoforms and that this interaction is mediated by the CSD in HP1 and the LxVxL HP1 interaction motif in KDM2A.

### KDM2A and HP1 form a complex binding circuit on chromatin

**Figure 3.**
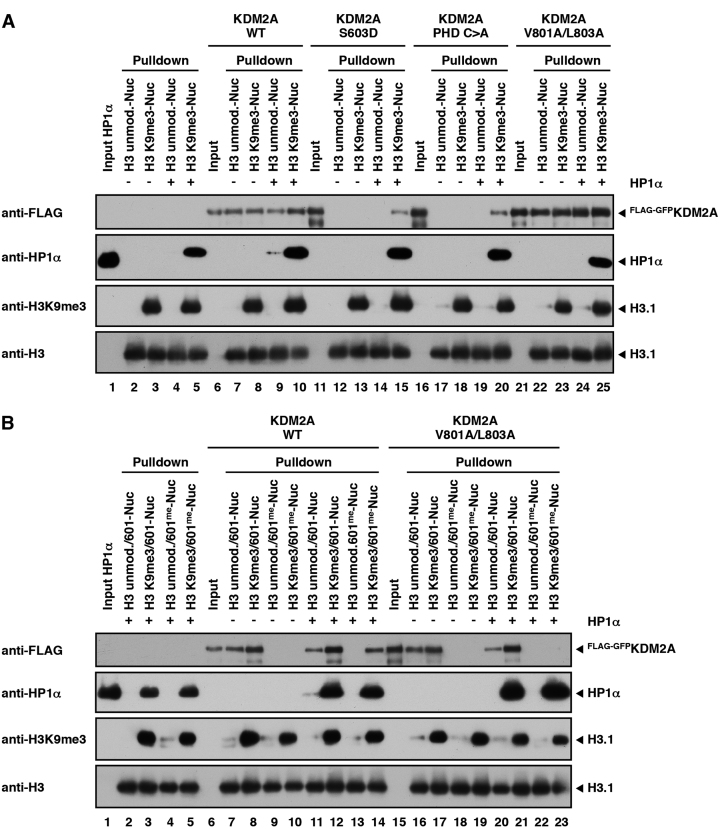
HP1α recruits KDM2A to H3K9me3-modified nucleosomes independently of DNA binding. (**A**) HP1-mediated recruitment of KDM2A to H3K9me3-modified nucleosomes independently of the ZnF and PHD domains. Unmodified or H3K9me3-modified nucleosomes were immobilized on streptavidin beads and incubated with WT HP1α and WT full length FLAG-GFP-tagged KDM2A or mutants in the ZnF (S603D) or PHD (PHD C>A) domains or the LxVxL motif (V801A/L803A) as indicated. 15% of the KDM2A inputs and pull-downs and 3% of the HP1α input were separated by SDS-PAGE and nucleosome-bound KDM2A and HP1α were detected by immunoblot. Equal loading was confirmed by immunoblot against Histone H3 and H3K9me3 modification of histone H3 was verified by immunoblot against the H3 tri-methyl lysine 9 mark. The apparent stronger binding of the V801A/L803A mutant is due to the difference in the input compared to the WT pull-downs. (**B**) Recruitment of KDM2A to H3K9me3-modified nucleosomes via HP1 is mediated through its LxVxL motif. Unmodified or H3K9me3-modified nucleosomes containing either unmethylated or CpG-methylated 601-DNA were immobilized on streptavidin beads and incubated with WT HP1α and WT full length FLAG-GFP-tagged KDM2A or the V801A/L803A mutant as indicated. Binding of KDM2A and HP1α to the nucleosomes was detected as described in Figure 3A.

The initial nucleosome binding experiments described above were carried out in protein extracts. In order to eliminate the influence of other factors present in the extracts on the interaction between KDM2A, HP1 and nucleosomes we performed nucleosome pull-downs with purified components using recombinant HP1α and purified full length FLAG-GFP-tagged KDM2A (Figure 3A, B and Supplementary Figure S4C). In this purified system nucleosome binding by wild-type KDM2A was independent of HP1α or the H3K9me3-modification (Figure 3A, lanes 7–10) most likely through direct contacts of the CXXC-ZnF with the linker DNA extending from the nucleosomes used in our assays for ∼20 bp on either side. Adding nuclear extract to the binding reactions resulted in a strong decrease in binding of recombinant KDM2A (Supplementary Figure S4A) indicating competition by other factors present in the extract for binding sites on the nucleosomes. Under these conditions KDM2A showed increased recruitment to H3K9me3-modified nucleosomes when HP1α was added to the binding reaction similar to overexpressed KDM2A in 293T extracts. This increase was not observed for the V801A/L803A mutant (Supplementary Figure S4B) demonstrating that in situations in which competition limits access of KDM2A to the nucleosomes the number of available binding sites between KDM2A and chromatin becomes critical for efficient recruitment. Using the inactivating point mutations in the ZnF (S603D) and the PHD domain (PHD C>A) in the purified system completely abolished nucleosome binding by KDM2A (Figure 3A, lanes 12, 13 and 17, 18) confirming that the PHD domain plays a crucial role in nucleosome recognition in addition to the CXXC-ZnF, which is known to mediate binding to CpG dinucleotides present in the linker DNA (6,7). The V801A/L803A mutant in the HP1 interaction motif retained the ability to bind to nucleosomes (Figure 3A, lanes 22 to 25) indicating that it is not directly involved in nucleosome recognition. Interestingly, both, the ZnF mutant and the PHD mutant could be recruited to H3K9me3-modified nucleosomes in the presence of HP1α (Figure 3A, lanes 15 and 20) pointing toward a DNA-independent recruitment mechanism of KDM2A to chromatin via HP1.

In order to test this hypothesis further we performed pull-down experiments with nucleosomes containing CpG-methylated DNA. As expected, CpG methylation abolished KDM2A binding to nucleosomes (Figure 3B, lanes 9 and 10). However, KDM2A could be recruited to H3K9me3-modified nucleosomes containing methylated DNA in the presence of HP1α (Figure 3B, lane 14). This binding was not detected when using the V801A/L803A mutant (Figure 3B, lane 23). These findings confirm a direct recruitment mechanism of KDM2A to chromatin via HP1 and that this binding is mediated through its LxVxL HP1 interaction motif.

**Figure 4.**
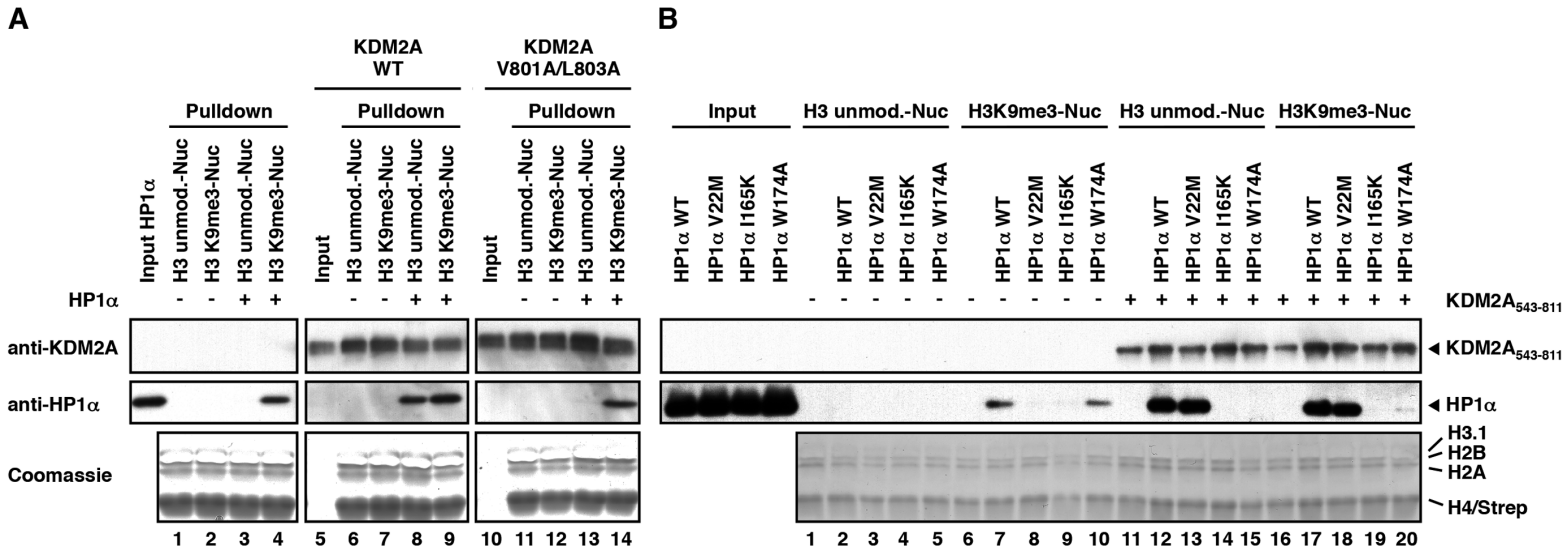
KDM2A mediates H3K9me3-independent binding of HP1α to nucleosomes. (**A**) KDM2A recruits HP1α to unmodified nucleosomes via the LxVxL motif. Unmodified or H3K9me3-modified nucleosomes were immobilized on streptavidin beads and incubated with WT HP1α and WT KDM2A_543-811_ or the V801A/L803A mutant as indicated. Fifteen percent of the KDM2A_543–811_ inputs and pull-downs and 3% of the HP1α input were separated by SDS-PAGE and nucleosome-bound KDM2A_543-811_ and HP1α were detected by immunoblot. Equal loading was confirmed by Coomassie stain. (**B**) Recruitment of HP1α to unmodified nucleosomes via KDM2A requires an intact chromo shadow domain. Nucleosome pull-downs were carried out as described in Figure 4A with WT KDM2A_543–811_ and WT or mutant HP1α (V22M, I165K or W174A mutants) and as indicated.

We also detected a signal for HP1α-binding to unmodified nucleosomes in the presence of KDM2A (Figure 3A, lane 9 and Figure 3B, lane 11). This binding was absent in the V801A/L803A mutant (Figure 3A, lane 24 and Figure 3B, lane 20) despite robust recruitment of this mutant to the nucleosomes. This finding could be confirmed using the recombinant KDM2A_543-811_ nucleosome recognition module described above. Nucleosome pull-downs using WT and V801A/L803A mutant KDM2A_543–811_ (Figure 4A), nucleosome pull-downs with WT HP1α and HP1α mutants carrying the inactivating mutations in the chromo (V22M) and chromo shadow (I165K, W174A) domains described above (Figure 4B), and titration experiments with the WT proteins (Supplementary Figure S5) demonstrate that KDM2A is able to recruit HP1 to chromatin in a H3K9me3-independent manner via a LxVxL/CSD interaction.

Interestingly, we also observed that while the HP1α W174A mutant in the CSD could not be recruited to unmodified nucleosomes through KDM2A (Figure 4B, lane 15) it was still able to bind to H3K9me3-modified nucleosomes on its own (Figure 4B, lane 10). This binding was not detected in the I165K mutant (Figure 4B, lane 9). In contrast to the I165K mutant, which is deficient in cofactor binding to the CSD and in HP1 dimerization, the W174A mutant is only deficient in cofactor binding but retains the ability to dimerize (30,33). This finding implies a crucial role for the dimerization of HP1 in nucleosome recognition.

Taken together these observations suggest a complex regulation of KDM2A binding to chromatin, which is modulated by both DNA methylation and the presence of H3K9 tri-methylation and HP1. Our data also demonstrate an H3K9me3-independent recruitment mechanism for HP1 through KDM2A.

**Figure 5.**
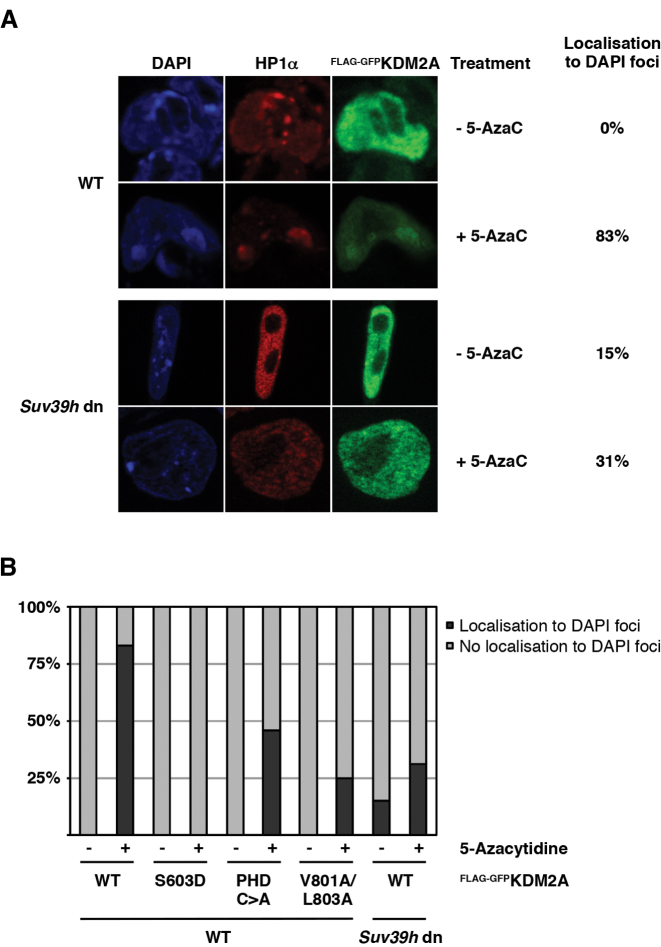
The interaction between KDM2A and HP1 is required for targeting of KDM2A to heterochromatic repeats *in vivo*. (**A**) KDM2A localizes to pericentromeric heterochromatin in the presence of HP1 when DNA is unmethylated. Mouse ES cells were transfected with a FLAG-GFP-tagged KDM2A expression construct and treated with 0.25 μM 5-Azacytidine (5-AzaC) for 24 hours to demethylate the DNA. The effectiveness of the 5-AzaC treatment was tested by restriction digest of genomic DNA (see Supplementary Figure S6). Localization of FLAG-GFP-KDM2A and endogenous HP1α to DAPI-stained heterochromatic foci was then detected by immunofluorescence. In untreated cells KDM2A shows diffuse staining throughout the nucleus. In 5-AzaC-treated WT ES cells, however, KDM2A co-localizes with HP1α in heterochromatic foci in the great majority of cells. This 5-AzaC-induced targeting of KDM2A to DAPI foci is reduced in *Suv39h* dn ES cells deficient for the H3K9 methyltransferases SUV39H1/2 in which HP1α does not localize to pericentromeric heterochromatin. (**B**) Targeting of KDM2A to pericentromeric heterochromatin requires interaction with HP1. WT and *Suv39h* dn mouse ES cells were transfected with FLAG-GFP-tagged KDM2A WT and mutant expression constructs and treated with 5-Azacytidine as indicated. The localization of FLAG-GFP-KDM2A and endogenous HP1α was then determined by immunofluorescence as in Figure 5A. Cells were counted and scored based on localization of KDM2A to DAPI-stained heterochromatic foci. The targeting of KDM2A to DAPI foci observed for the WT in the majority of 5-AzaC-treated WT ES cells is absent in the S603D mutant and strongly reduced in the PHD C>A and V801A/L803A mutants. Heterochromatic localization of WT KDM2A is reduced to similar levels in *Suv39h* dn ES cells. The effectiveness of the 5-AzaC treatment was tested by restriction digest of genomic DNA (see Supplementary Figure S6).

The ability of both proteins to bridge between nucleosomes and the respective other factor results in the formation of a complex recruitment circuit on chromatin. To demonstrate that this binding loop is also operational on native chromatin *in vivo* we made use of the observation that KDM2A preferentially localises to DAPI dense pericentromeric heterochromatin foci in mouse cells that are devoid of DNA methylation (7). We expressed FLAG-GFP-tagged WT KDM2A or the ZnF, PHD or HP1-binding motif mutants in wild-type mouse ES cells that were either left untreated or treated with the DNMT1-inhibitor 5-Azacytidine to reduce the levels of DNA methylation (Supplementary Figure S6). We then analysed the nuclear localization of KDM2A by immunofluorescence against the GFP-tag (Figure 5A and B). In untreated cells all constructs showed a diffuse nuclear distribution due to high DNA methylation levels. In 5-Azacytidine-treated cells, however, WT KDM2A was found to localize to DAPI dense heterochromatin foci in the vast majority of cells similar to the localization observed in *DNMT1^−/−^* mouse embryonic fibroblasts (7). This localization to DAPI foci was not observed for the S603D mutant confirming that the recognition of unmethylated CpGs by the ZnF is critically important for KDM2A to localize to the pericentromeric repeats (Figure 5B). Importantly the localization of the HP1 binding-deficient V801A/L803A mutant to the pericentromeric heterochromatin was strongly reduced in the 5-Azacytidine-treated cells (Figure 5B). The mutant in the PHD finger also showed reduction in recruitment although not to the extent seen in the V801A/L803A mutant. Furthermore, 5-Azacytidine treatment of *Suv39h* dn ES cells in which both genes (*Suv39h1* and *Suv39h2*) coding for the H3K9-methyltransferase SUV39H are deleted (17) did not result in a dramatic increase of localization of WT KDM2A to DAPI foci as seen in the wild type cells. The *Suv39h* dn ES cells show reduced DNA methylation and lack H3K9 tri-methylation at pericentromeric heterochromatin and as a consequence HP1 proteins are no longer found at the pericentromeric repeats (34) (Figure 5A). These results confirm that also *in vivo* KDM2A preferentially localises to H3K9me3- and HP1-marked chromatin when DNA methylation levels are low and that both conditions need to be met for effective targeting. In addition to the CXXC-ZnF that targets KDM2A to unmethylated GpGs, the interaction between KDM2A and HP1 is critical for this recruitment.

**Figure 6.**
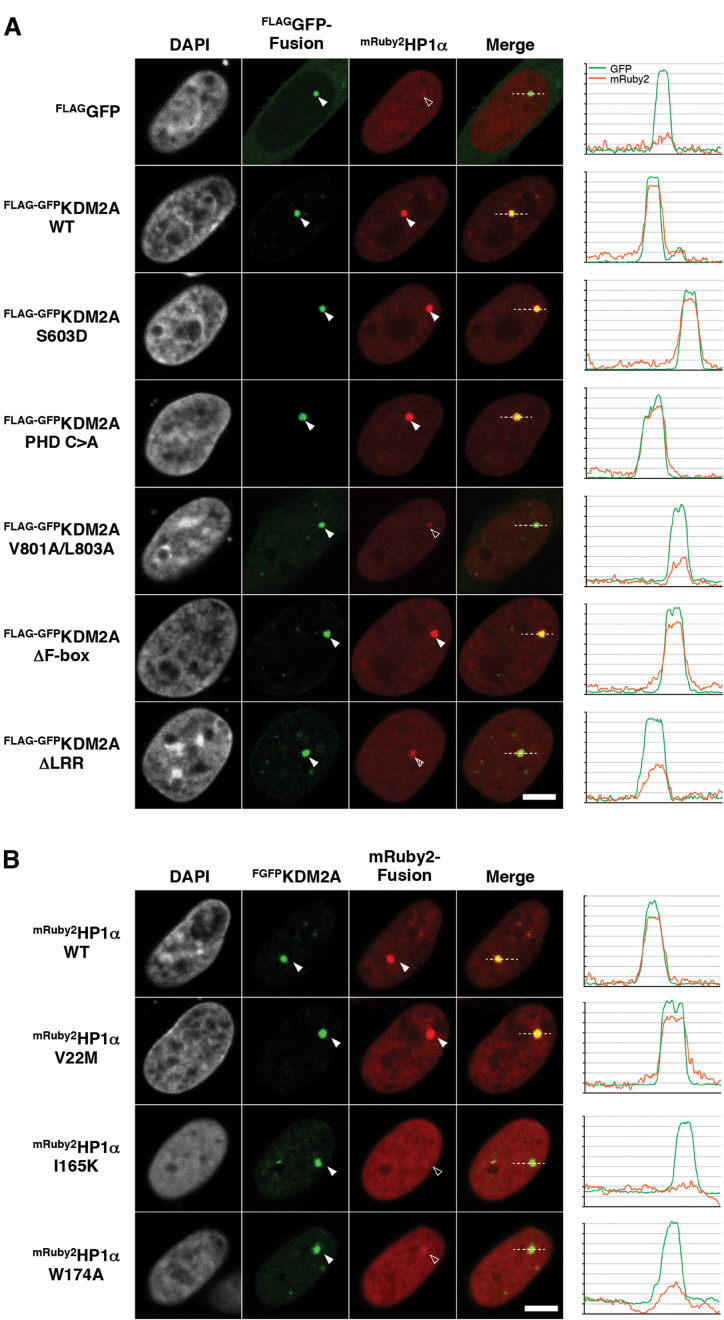
KDM2A can tether HP1 to chromatin *in vivo*. (**A**) Analysis of the KDM2A-mediated recruitment of HP1α to chromatin in a cell-based F3H assay. FLAG-GFP-tagged WT KDM2A and different mutants were expressed together with mRuby2-fused HP1α and a GBP-LacI anchor construct in BHK cells harbouring a genomic *lac*O array. The GBP-LacI fusion protein anchors the GFP tagged KDM2A mutants to the *lac*O array and shows a green spot in the nucleus (second column, filled arrow head). mRuby2-fused HP1α was recruited to the *lac*O spot according to its interaction affinity with the respective KDM2A mutants (third column, arrowhead). The KDM2A V801A/L803A mutation shows a significant reduction of the interaction with HP1α. Line intensity profiles of the GFP and mRuby2 are shown next to the image. Scale bar equals to 5 μm. Quantifications of HP1 binding relative to KDM2A binding can be found in Supplementary Figure S7C. (**B**) Test of the interaction between KDM2A and HP1α point mutants. While the V22M mutation of HP1α has no obvious impact on the interaction (third column, filled arrowhead), the I165K and W174A mutations abolish the interaction between HP1α and KDM2A (third column, open arrowhead). For quantifications see Supplementary Figure S7D.

Furthermore, we could demonstrate that KDM2A is able to tether HP1 proteins to chromatin *in vivo* by making use of a cell-based fluorescent-three-hybrid (F3H) assay (20) (Figure 6A, B and Supplementary Figure S7A-D). For this assay FLAG-GFP-tagged KDM2A and red fluorescent protein (mRuby2) fused HP1 were co-expressed in BHK cells and KDM2A was targeted to a *lac* operator (*lac*O) array inserted into the genome by a co-transfected GBP-LacI anchor. Localization of GFP-KDM2A and mRuby2-HP1 to the *lac*O array was then detected by fluorescence microscopy. Using the various KDM2A and HP1 constructs described above confirms that KDM2A is able to recruit all HP1 isoforms to chromatin also *in vivo* (Supplementary Figure S7A and S7D) and that the interaction is mediated by the HP1-binding motif in KDM2A (Figure 6A and Supplementary Figure S7C) and the CSD in HP1 (Figure 6B and Supplementary Figure S7D).

### The HP1 binding motif is conserved and enriched for cancer mutations

**Figure 7.**
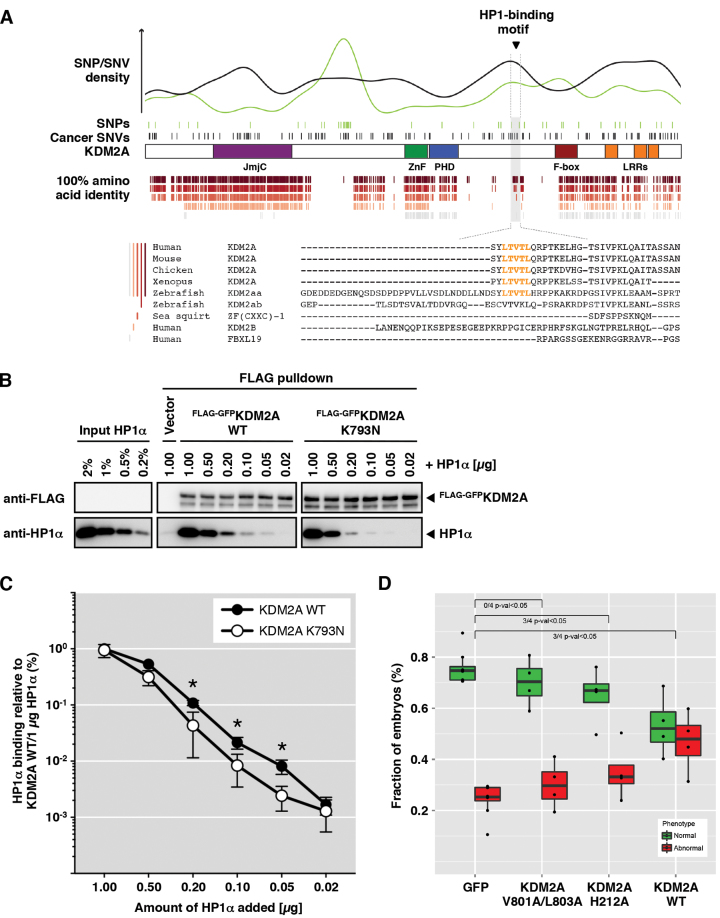
The HP1 interaction is integral to the biological function of KDM2A. (**A**) Domain-specific patterns of conservation and single nucleotide variations in KDM2A. Missense variants segregating in the human population (SNPs, green ticks) or present in a collection of sequenced tumours (cancer SNVs, black ticks) are indicated in relation to the linear domain architecture of human KDM2A. Correspondingly coloured density plots highlight the anti-correlated incidence of SNPs and cancer SNVs across KDM2A domains (SNP/SNV density). Identical amino acids across different subsets of KDM2A orthologs/gene family members are illustrated below for individual residues (ticks). A Sequence alignment of the region surrounding the HP1 binding motif (LTVTL) demarcated by dotted lines indicates conservation of the motif in KDM2A orthologues but not KDM2B and Fbxl19. A full alignment of KDM2A orthologues spanning the whole protein sequence is provided in Supplementary File 1. (**B**) The K793N cancer mutation impairs binding of HP1α to KDM2A. FLAG-GFP-tagged KDM2A WT or the K793N cancer mutant were expressed in 293T cells and captured on FLAG-affinity beads. The immobilized KDM2A proteins were then incubated with different amounts of recombinant HP1α as indicated. 40% of the pull-down reactions were resolved by SDS-PAGE and bound proteins detected by immunoblot. Titration of HP1α indicates reduced binding to the K793N mutant compared to KDM2A WT. (**C**) Quantification of HP1 binding to WT KDM2A and the K793N mutant. The FLAG and HP1α immunoblots shown in Figure 7B were quantified using the chemiluminescence signals associated with the full length FLAG-GFP-KDM2A and HP1α bands. HP1α signals were normalized against the WT KDM2A pull-down with 1 μg HP1α using the FLAG (KDM2A) signals obtained for the individual pull-downs as a relative measure for loading. The figure shows the mean and standard deviation of three independently performed experiments plotted on a logarithmic (log_10_) scale. Asterisks mark samples in which binding of HP1α to the K793N mutant differs significantly from the WT (*P*-value < 0.05 by unpaired *t*-test). The K793N mutant shows ∼50% reduced binding over a wide range of HP1α concentrations. (**D**) Loss of function of the KDM2A V801A/L803A mutant. Box plot of overexpression phenotypes caused by injecting 3.6 nl of 62.5 ng/μl mRNA of GFP and three human KDM2A constructs into zebrafish embryos shows that there was no significant difference in phenotype between GFP and V801A/L803A mutant KDM2A, whereas in three out of four injections both H212A mutant KDM2A and WT KDM2A showed significantly more abnormal embryos at 24 h.p.f. than the GFP control. Statistical analyses of the individual overexpression experiments can be found in Supplementary Figure S9C–E.

In search of evidence for a biological significance of the HP1 interaction we performed database searches for single nucleotide polymorphisms (SNPs) and disease-associated point mutations in KDM2A. Disease-associated single nucleotide variants (SNVs) resulting in harmful amino acid exchanges are often found concentrated in functionally important domains of their gene products. Computational analysis of missense SNVs in KDM2A deposited in the public COSMIC (24) and cBioPortal/TCGA (25,26) cancer mutation databases indeed indicates an accumulation of cancer-associated SNVs in the region of the HP1 binding motif (Figure 7A and Supplementary Table S1). The mutation density over the HP1 motif is similar to the one observed over the catalytic centre of the JmjC demethylase domain. Comparison of the peptide sequences of KDM2A orthologues in different species further indicates that the HP1 interaction motif is perfectly conserved in vertebrates. This conservation is not observed in KDM2B and Fbxl19, close paralogues of KDM2A that differ significantly in their peptide sequences in the region between the PHD domain and the F-box (Figure 7A and Supplementary File 1). The high cancer mutation density and the evolutionary conservation of the HP1 binding motif suggest an important role for the HP1 interaction in the biological function of KDM2A alongside the H3K36 demethylase activity. To test whether any of the cancer mutations affect the interaction between KDM2A and HP1 we introduced several mutations found in the vicinity of the HP1 binding motif into our FLAG-GFP-KDM2A expression construct. These mutants were then expressed in 293T cells, immobilized on FLAG-affinity beads, and used in quantitative pull-down assays to compare their binding to recombinant HP1α relative to WT KDM2A. This screen revealed that some mutations just N-terminal of the motif indeed impair the interaction between KDM2A and HP1 (Supplementary Figure S8A and S8B). The strongest effect was observed for the K793N mutant found in intestinal cancer which showed ∼50% reduced HP1 binding compared to WT KDM2A (Figure 7B and C). These mutations appear to modulate and not completely disrupt the interaction between KDM2A and HP1 suggesting a deregulation rather than loss of function of KDM2A caused by certain cancer mutations.

### Inactivating the HP1 interaction motif abolishes the KDM2A overexpression phenotype in zebrafish embryos

As shown before KDM2A preferentially localizes to heterochromatic foci in cells with reduced levels of DNA methylation (7). In addition, KDM2A knock out mice display embryonic lethality, which demonstrates an important function for KDM2A in embryonic development (35). Early embryonic cell divisions are characterized by large-scale genomic demethylation events and re-establishment of tissue-specific DNA methylation patterns during differentiation processes (36). The interaction between KDM2A and HP1 might, therefore, be relevant during certain ‘windows of opportunity’ in early developmental processes when the genome is unmethylated. We therefore sought an experimental system in which the impact of the HP1 interaction on the function of KDM2A could be tested during early embryonic development *in vivo*. For this, zebrafish is a suitable model organism as embryos develop outside the body and the effects of the expression of exogenous proteins on early embryonic development can be readily observed. To test the relevance of the HP1 interaction for KDM2A function we therefore overexpressed wild-type and mutant versions of FLAG-GFP-tagged human KDM2A by injecting mRNA into one-cell stage wild-type zebrafish embryos. First we confirmed expression of all constructs by scoring GFP fluorescence at the dome stage (Supplementary Figure S9A). We then tested for an overexpression phenotype by injecting 3.6 nl of 62.5 ng/μl mRNA from each construct into at least 150 embryos with GFP mRNA as the control (Figure 7D and Supplementary Figure S9C–E). This was repeated four times for each construct. We scored the embryos for phenotypic abnormalities (Supplementary Figure S9B) at 24 h post fertilization (h.p.f.) after excluding any embryos that were unfertilized or appeared damaged on the day of the injection. We found a significant increase in the number of embryos displaying an abnormal phenotype in three out of four injections for both the wild-type KDM2A and the JmjC demethylase-inactivating H212A mutant compared to the GFP control (Figure 7D). By contrast, there was no statistically significant difference between the GFP control and the V801A/L803A mutant with the inactivated HP1 interaction motif. This demonstrates that in this *in vivo* overexpression assay the HP1 interaction is essential for the function of KDM2A, while the demethylase activity is largely dispensable. This strongly suggests that KDM2A functions to a large extent through its interaction with HP1 proteins.

## DISCUSSION

DNA and histone modifications are fundamental to the regulation of gene expression and the structural organization of chromosomes. Together, these modifications create distinct local chromatin environments that are interpreted by epigenetic factors. In order to integrate information from multiple modifications epigenetic readers must be able to engage in multivalent interactions with multiple binding sites on chromatin. In this study we have used *in vitro* biochemical and cell-based approaches to dissect how the histone H3K36 demethylase KDM2A decodes different nucleosomal DNA and histone modification states.

Like many epigenetic factors KDM2A is a highly modular protein with a complex domain structure. Based on our previous findings that KDM2A can recognize H3K9me3-modified chromatin via HP1 proteins (3) we have mapped the domains in KDM2A that are necessary for chromatin binding. We have identified an extended chromatin recognition module within KDM2A that allows it to read out the nucleosomal DNA and histone modification status. This ‘nucleosome interaction module’ spans from the CXXC-ZnF to a newly identified variant HP1 interaction motif downstream of the PHD domain that enables KDM2A to directly bind to HP1. While it has been established that the CXXC-ZnF mediates binding to unmodified CpG dinucleotides (6,7) we also find that the PHD domain is necessary for nucleosome recognition by KDM2A. Whether the PHD domain is directly involved in nucleosome contacts or whether it provides structural support for other parts of the protein remains to be determined. Our experiments show that the direct interaction of KDM2A with CpG dinucleotides and HP1 enables complex binding dynamics between both factors on chromatin that are modulated by DNA- and H3K9-methylation. The multivalent interaction leads to preferential targeting of KDM2A to H3K9me3-modified chromatin that is decorated with HP1 and contains unmethylated CpGs, and to reduced binding when the DNA is methylated. Importantly, the presence of KDM2A on chromatin containing unmethylated DNA also enables recruitment of HP1 in an H3K9me3-independent manner. Evolutionary conservation and an enrichment of cancer-associated single nucleotide variations that potentially interfere with the KDM2A/HP1 interaction indicate an important biological function for the HP1 binding motif. Mutating the LxVxL motif leads to loss of activity of KDM2A in an *in vivo* overexpression assay in zebrafish embryos, presumably through disrupting the interaction with endogenous zebrafish HP1 proteins, demonstrating that HP1 binding plays a major role for the function of KDM2A.

**Figure 8.**
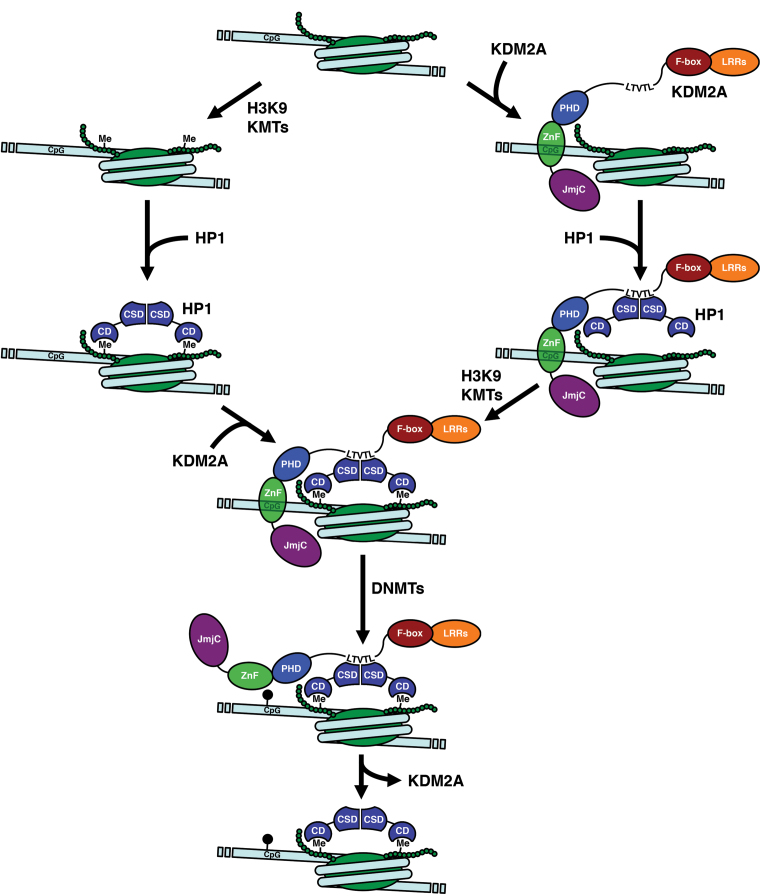
Model for KDM2A-mediated establishment of heterochromatin. The CXXC-Znf enables KDM2A to bind chromatin containing no pre-existing DNA and histone modifications (top). Binding of HP1 to the KDM2A LTVTL motif establishes an alternative to the HP1 recruitment mechanism via the H3K9me3-modification deposited by H3K9 lysine methyltransferases (KMTs) that does not require the K3K9me3 mark. Subsequent deposition of the H3K9me3-modification by H3K9 KMTs leads to reinforced HP1 binding and a stabilization of the KDM2A/HP1/nucleosome interaction. The ability of HP1 to recruit DNMTs leads to deposition of CpG methylation (black pinheads) which results in loss of KDM2A leaving behind DNA-methylated, H3K9me3-modified and HP1-decorated heterochromatin (bottom).

KDM2A has been shown to be involved in the silencing of centromeric satellite repeats (8) and of rRNA genes (5,9). The ability to directly recruit HP1 to unmodified chromatin suggests a function for KDM2A as an important factor in the *de novo* establishment or maintenance of silenced chromatin (Figure 8). Indeed, KDM2A preferentially localises to pericentromeric heterochromatin when DNA is unmethylated (7) and loss of KDM2A results in disruption of heterochromatin (8). The CXXC-ZnF allows KDM2A to be targeted to ‘naïve’ chromatin regions containing unmethylated CpG dinucleotides and no pre-existing repressive histone modifications. In addition to erasing H3K36 methylation KDM2A could act as a landing platform for HP1, which in turn would result in the recruitment of H3K9-methyl transferases, leading to deposition of H3K9-methylation and a reinforcement of HP1 binding. Subsequent recruitment of DNMTs (27,28) through HP1 would result in the deposition of CpG-methylation on the DNA, which would lead to the destabilization of the KDM2A/DNA interaction and ultimately eviction of KDM2A. KDM2A-mediated recruitment of HP1 might, therefore, be the transient initial trigger for a feedback loop resulting in establishment of H3K9 and CpG methylation to form ‘mature’ heterochromatin. Despite the overall similarity in peptide sequence between KDM2A and its close paralogue KDM2B this ability to recruit HP1 to naïve chromatin is most likely limited to KDM2A since the HP1 interaction motif is not conserved in KDM2B.

Taken together, our experiments show a complex regulation of nucleosome binding by KDM2A and that a direct interaction between KDM2A and HP1 is integral to its function. Through this interaction KDM2A has the potential to be directly involved in heterochromatin regulation. Defects in establishing and maintaining heterochromatin would result in transcriptional deregulation and genomic instabilities that could contribute to the cancerous phenotypes observed in cells with aberrant KDM2A expression (8,10–14). Although the exact contribution of the HP1 interaction to the *in vivo* role of KDM2A remains to be established, it is becoming evident that KDM2A has important functions beyond its histone demethylase activity. Recent advances in genome engineering technologies will enable a detailed analysis of its various activities by introducing inactivating point mutations into its functional domains and studying the effects in *in vivo* model systems. These studies will be key to unraveling how the individual activities contribute to the function of KDM2A in chromatin regulation and development, and shed light on the role of KDM2A in cancer formation.

## Supplementary Material

Supplementary Data
