# Supplementary material for: KDM2A integrates DNA and histone modification signals through a CXXC/PHD module and direct interaction with HP1
Source: Nucleic Acids Res. 2016 Oct 24;45(3):1114–29. doi: 10.1093/nar/gkw979 (PMC5388433; doi:10.1093/nar/gkw979)
Supplement: Supplementary Data [file gkw979_Supp.zip › nar-00658-m-2016-File010.pdf]

SUPPLEMENTARY MATERIAL

KDM2A integrates DNA and histone modification signals through a CXXC/PHD module and direct interaction with HP1

Julie Borgel, Marek Tyl, Karin Schiller, Zsofia Pusztai, Christopher M. Dooley, Wen Deng, Carol Wooding, Richard J. White, Tobias Warnecke, Heinrich Leonhardt, Elisabeth M. Busch-Nentwich and Till Bartke

SUPPLEMENTARY FIGURES

Supplementary Figure S1. Recruitment of KDM2A to H3K9me3-modified nucleosomes is stimulated by HP1 and counteracted by CpG-methylation, related to Figure 1.

Supplementary Figure S2. Mapping of nucleosome binding sites within KDM2A, related to Figure 1.

Supplementary Figure S3. Size exclusion chromatography of complexes formed between the KDM2A nucleosome recognition module and HP1α CSD mutants and the HP1 isoforms β and γ, related to Figure 2.

Supplementary Figure S4. HP1α recruits KDM2A to H3K9me3-modified nucleosomes independently of DNA binding, related to Figure 3.

Supplementary Figure S5. KDM2A mediates H3K9me3-independent binding of HP1α to nucleosomes, related to Figure 4.

Supplementary Figure S6. The interaction between KDM2A and HP1 is required for targeting of KDM2A to heterochromatic repeats *in vivo*, related to Figure 5.

Supplementary Figure S7. KDM2A interacts with all HP1 isoforms *in vivo*, related to Figure 6.

Supplementary Figure S8. KDM2A cancer mutations N-terminal of the HP1 binding motif impair the interaction between KDM2A and HP1α, related to Figure 7.

Supplementary Figure S9, Expression of FLAG-GFP-tagged KDM2A constructs in zebrafish embryos and overexpression phenotype, related to Figure 7.

SUPPLEMENTARY MATERIALS AND METHODS

SUPPLEMENTARY FILES

Supplementary File 1: KDM2A Sequence Alignments (PDF), Related to Figure 7

SUPPLEMENTARY TABLES

Supplementary Table 1: SNPs and cancer SNVs identified in KDM2A (Excel file), related to Figure 7

SUPPLEMENTARY REFERENCES

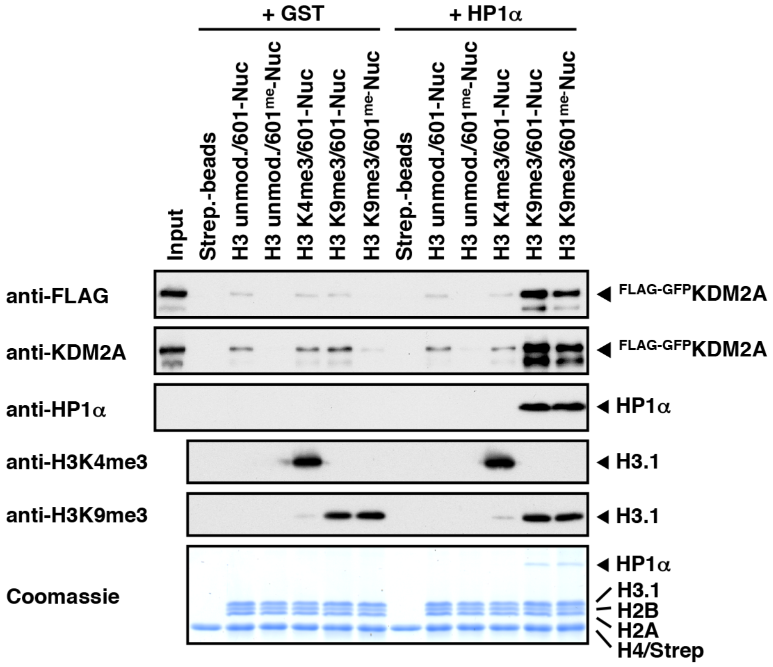

Supplementary Figure S1. Recruitment of KDM2A to H3K9me3-modified nucleosomes is stimulated by HP1 and counteracted by CpG-methylation, related to Figure 1.

293T whole cell extracts overexpressing FLAG-GFP-tagged KDM2A were incubated with immobilised modified nucleosomes as indicated. Binding reactions were supplemented with recombinant purified HP1α or GST as a control. 40% of each input and pull-down were separated by SDS-PAGE and nucleosome-bound FLAG-GFP-KDM2A and HP1α were detected by immunoblot. Equal loading was confirmed by Coomassie stain and modification of histone H3 was verified by immunoblot against H3 tri-methyl lysine marks.

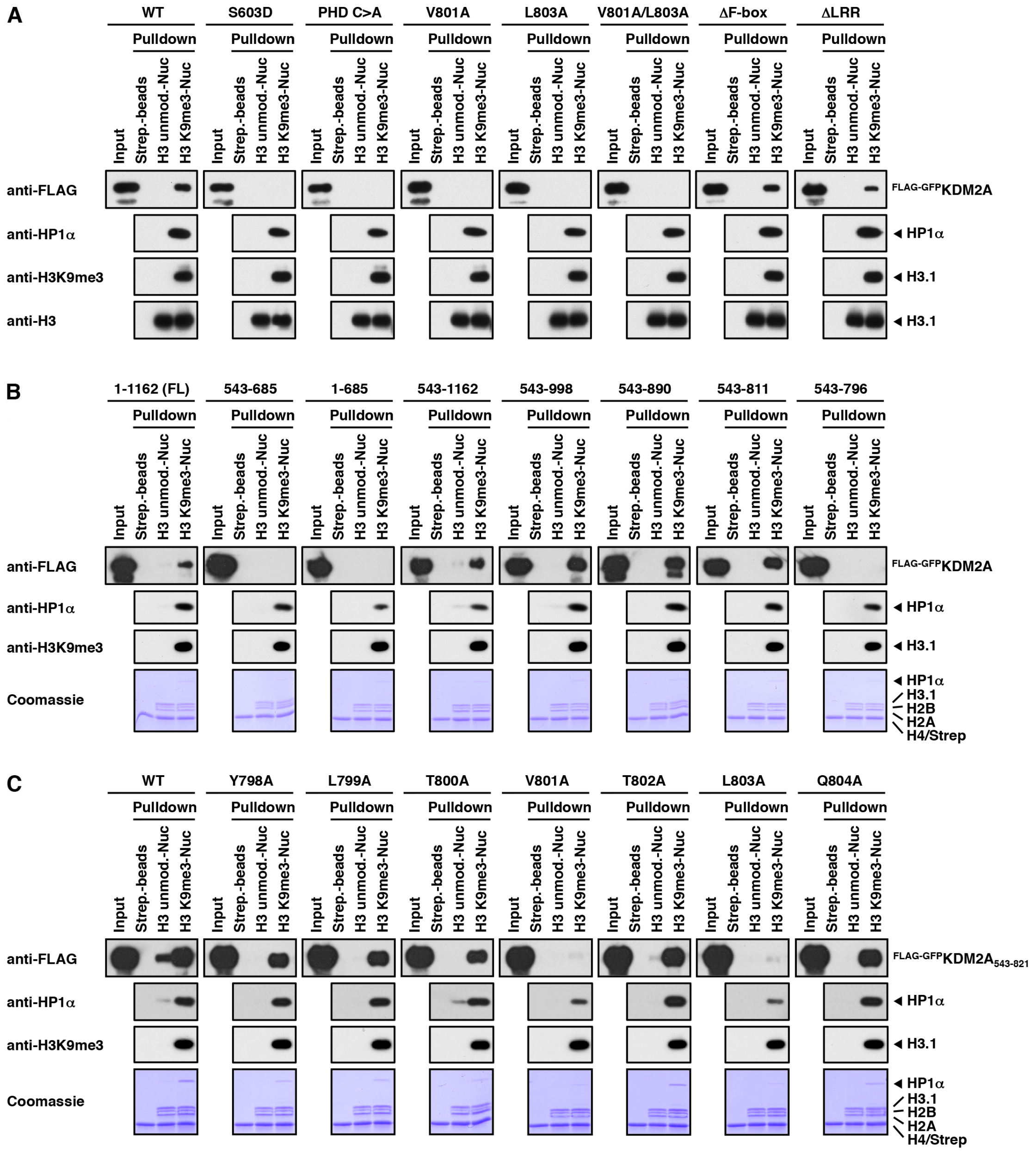

Supplementary Figure S2. Mapping of nucleosome binding sites within KDM2A, related to Figure 1.

(A) Mapping of nucleosome binding domains in KDM2A. Unmodified or H3K9me3-modified nucleosomes were immobilised on streptavidin beads and incubated with 293T whole cell extracts overexpressing wild-type (WT) FLAG-GFP-tagged KDM2A or point/deletion mutants as indicated. All binding

reactions were supplemented with recombinant purified HP1α. 40% of each input and pull-down were separated by SDS-PAGE and nucleosome-bound FLAG-GFP-KDM2A and HP1α were detected by immunoblot. Equal loading was confirmed by immunoblot against Histone H3 and H3K9me3 modification of histone H3 was verified by immunoblot against the H3 tri-methyl lysine 9 mark. This figure is an extended version of Figure 1C including all controls.

(B) Mapping of a nucleosome interaction module within KDM2A. Nucleosome binding reactions were carried out as described above using FLAG-GFP-tagged full length (FL) KDM2A or N- or C-terminal deletion mutants including amino acids as indicated. All binding reactions were supplemented with recombinant purified HP1α. 40% of each input and pull-down were separated by SDS-PAGE and nucleosome-bound FLAG-GFP-KDM2A and HP1α were detected by immunoblot. Equal loading was confirmed by Coomassie stain and H3K9me3 modification of histone H3 was verified by immunoblot against the H3 tri-methyl lysine 9 mark. This figure is an extended version of Figure 1D including all controls.

(C) Mapping of a HP1-binding site within the KDM2A nucleosome interaction module. Unmodified or H3K9me3-modified nucleosomes were immobilised on streptavidin beads and incubated with 293T whole cell extracts overexpressing a FLAG-GFP-tagged KDM2A fragment spanning amino acids 543 to 821 and containing single alanine point mutations of amino acids 798 to 804 as indicated. All binding reactions were supplemented with recombinant purified HP1α. 20% of each input and pull-down were separated by SDS-PAGE and nucleosome-bound FLAG-GFP-KDM2A and HP1α were detected by immunoblot. Equal loading was confirmed by Coomassie stain and H3K9me3 modification of histone H3 was verified by immunoblot against the H3 tri-methyl lysine 9 mark.

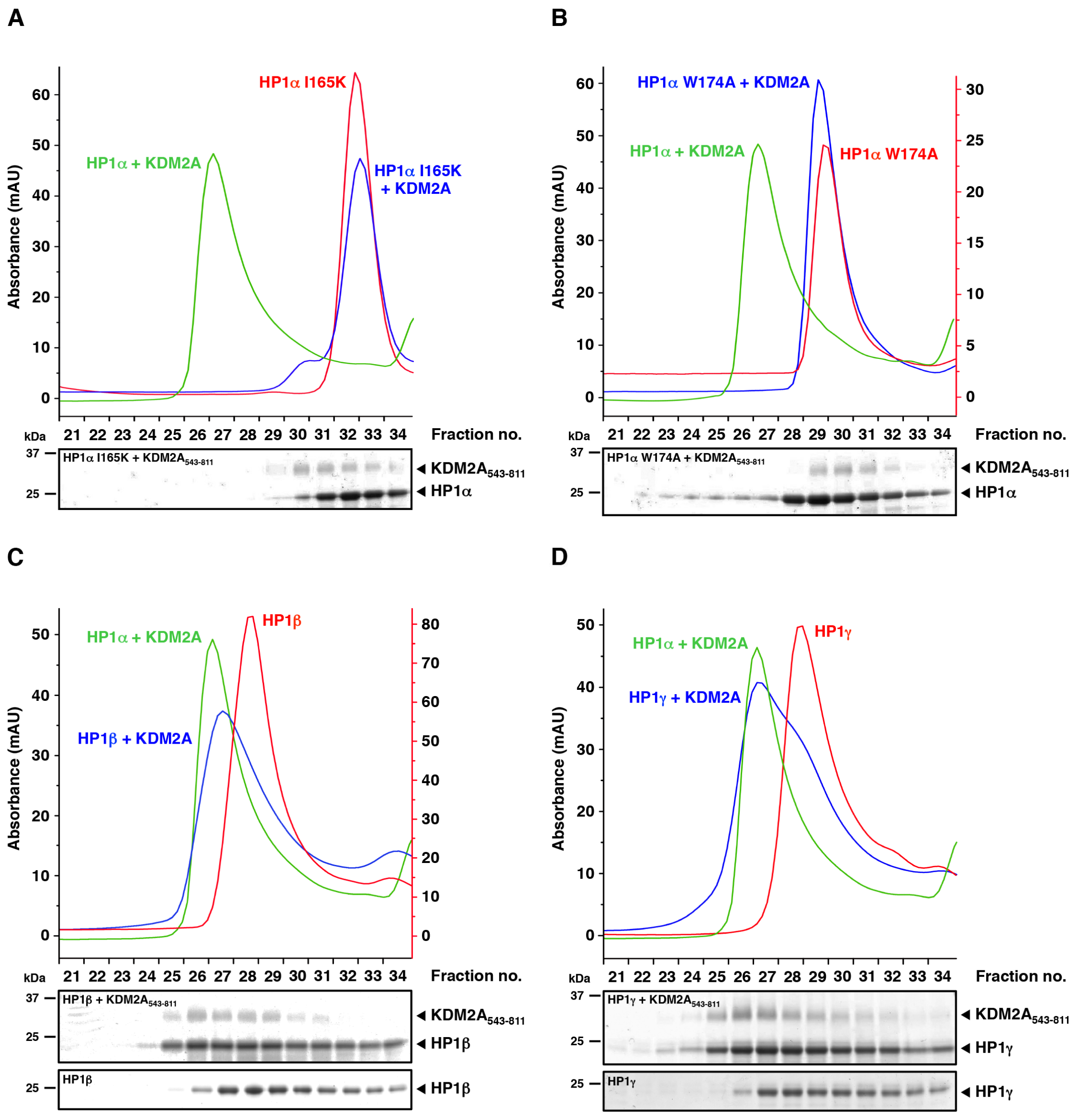

Supplementary Figure S3. Size exclusion chromatography of complexes formed between the KDM2A nucleosome recognition module and HP1α CSD mutants and the HP1 isoforms β and γ, related to Figure 2.

(A) The recombinant purified HP1α I165K mutant and the purified KDM2A nucleosome recognition module ($KDM2A_{543\text{-}811}$) were mixed and then separated by size exclusion chromatography on a Superdex 200 column (blue trace). Comparison with the chromatogram of the complex between the WT HP1α and $KDM2A_{543\text{-}811}$ proteins (green trace) indicates that no complex has

formed. Size exclusion chromatography of the HP1α I165K mutant alone (red trace) and comparison with the migration behaviour of WT HP1α (Figure 2D) further confirms that the I165K mutation in the CSD disrupts dimerisation of HP1α as described (1).

(B) Size exclusion chromatography of the purified HP1α W174A mutant and $KDM2A_{543\text{-}811}$ (blue trace) indicates that the W174A mutation in the CSD interferes with complex formation. The chromatogram of the complex between the WT proteins is shown for comparison (green trace). The migration behaviour of the HP1α W174A mutant alone (red trace) confirms that this mutant forms dimers similar to WT HP1α as described (1,2).

(C) Size exclusion chromatography of the complex formed between purified HP1β and $KDM2A_{543\text{-}811}$ indicates that the beta isoform of HP1 directly interacts with KDM2A (blue trace). The chromatogram of the complex between HP1α and $KDM2A_{543\text{-}811}$ is shown for comparison (green trace).

(D) Size exclusion chromatography of the complex formed between purified HP1γ and $KDM2A_{543\text{-}811}$ indicates that the gamma isoform of HP1 directly interacts with KDM2A (blue trace). The chromatogram of the complex between HP1α and $KDM2A_{543\text{-}811}$ is shown for comparison (green trace).

Borgel et al. Supplementary Figure S4

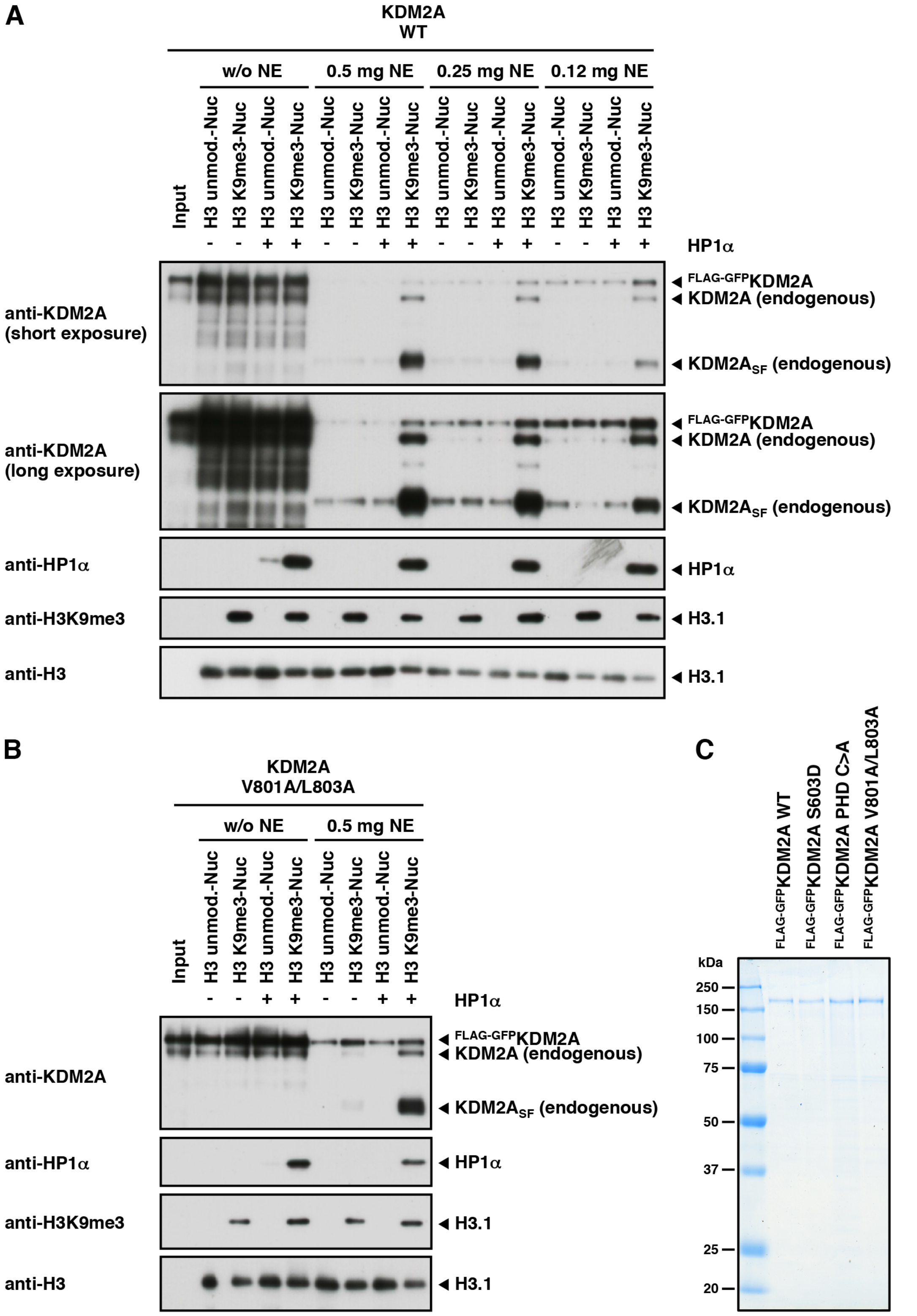

Supplementary Figure S4. HP1α recruits KDM2A to H3K9me3-modified nucleosomes independently of DNA binding, related to Figure 3.

(A) Nucleosome binding assays with purified full length KDM2A in the presence of nuclear extract. Unmodified or H3K9me3-modified nucleosomes were immobilised on streptavidin beads and incubated with WT HP1α and WT full length FLAG-GFP-tagged KDM2A in the presence of increasing amounts of HeLaS3 nuclear extract as indicated. 15% of each input and pull-down were separated by SDS-PAGE and nucleosome-bound KDM2A and HP1α were detected by immunoblot. Equal loading was confirmed by immunoblot against Histone H3 and H3K9me3 modification of histone H3 was verified by immunoblot against the H3 tri-methyl lysine 9 mark. Proteins present in the nuclear extract compete with KDM2A and strongly reduce its binding to nucleosomes. Under these conditions addition of exogenous HP1α stimulates binding of the purified FLAG-GFP-KDM2A to H3K9me3-modified nucleosomes along with the endogenous full length KDM2A and $KDM2A_{SF}$ and similar to the experiments performed with overexpressed FLAG-GFP-tagged KDM2A in 293T extracts (Figures 1, S1 and S2).

(B) Nucleosome binding assays with purified full length KDM2A V801A/L803A in the presence of nuclear extract. Nucleosome binding assays were carried out with full length FLAG-GFP-tagged KDM2A V801A/L803A in the presence of HeLaS3 nuclear extract as described for Figure S4A. Competition with proteins present in the nuclear extract strongly reduces binding of KDM2A V801A/L803A to nucleosomes. Contrary to WT KDM2A no stimulation of binding to H3K9me3-modified nucleosomes by the addition of HP1α is observed for the V801A/L803A mutant.

(C) Purified full length FLAG-GFP-KDM2A proteins used in nucleosome binding reactions in Figures 3, S4A and S4B. FLAG-GFP-KDM2A WT and the S603D, PHD C>A, and V801A/L803A mutants were expressed in 293T cells and purified by FLAG-affinity chromatography. Approximately 1 μg of each protein was separated on a SDS-PAGE gel as indicated and purity was confirmed by Coomassie staining.

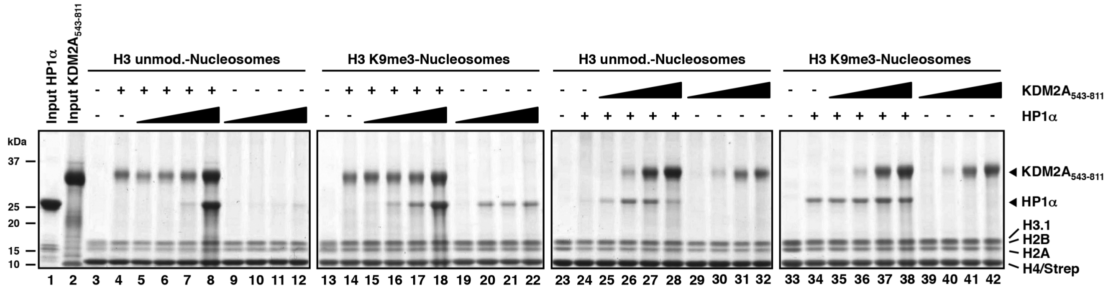

Supplementary Figure S5. KDM2A mediates H3K9me3-independent binding of HP1α to nucleosomes, related to Figure 4.

Titration of recombinant HP1α and KDM2A over unmodified and H3K9me3-modified nucleosomes. Nucleosomes were immobilised on streptavidin beads and incubated with increasing amounts of HP1α or $KDM2A_{543-811}$ in the presence of either a constant amount of $KDM2A_{543-811}$ (lanes 4 - 8 and 14 - 18) or HP1α (lanes 24 - 28 and 34 - 38), as indicated. 1.5 μg of the $KDM2A_{543-811}$ and HP1α inputs and 50% of the pull-down reactions were separated by SDS-PAGE and bound proteins were visualised by Coomassie staining. The KDM2A fragment binds to nucleosomes independently of HP1α (lanes 4 and 14) and binding is stimulated by an excess of HP1α (lanes 8 and 18). HP1α binds unmodified nucleosomes to the same extent as H3K9me3-modified nucleosomes in the presence of $KDM2A_{543-811}$ (lanes 8 and 18 and lanes 26 and 34) indicating that KDM2A recruits HP1α to nucleosomes independently of H3K9me3. In the presence of $KDM2A_{543-811}$ the binding of HP1α to H3K9me3-modified nucleosomes is strongly increased (compare lanes 18 and 22) demonstrating that KDM2A and HP1α show mutual enhancement of binding to H3K9me3-modified nucleosomes. This enhancement is only observed if HP1α is present at a ~2-fold excess over KDM2A suggesting a requirement for a particular stoichiometry between HP1α and KDM2A for efficient nucleosome binding.

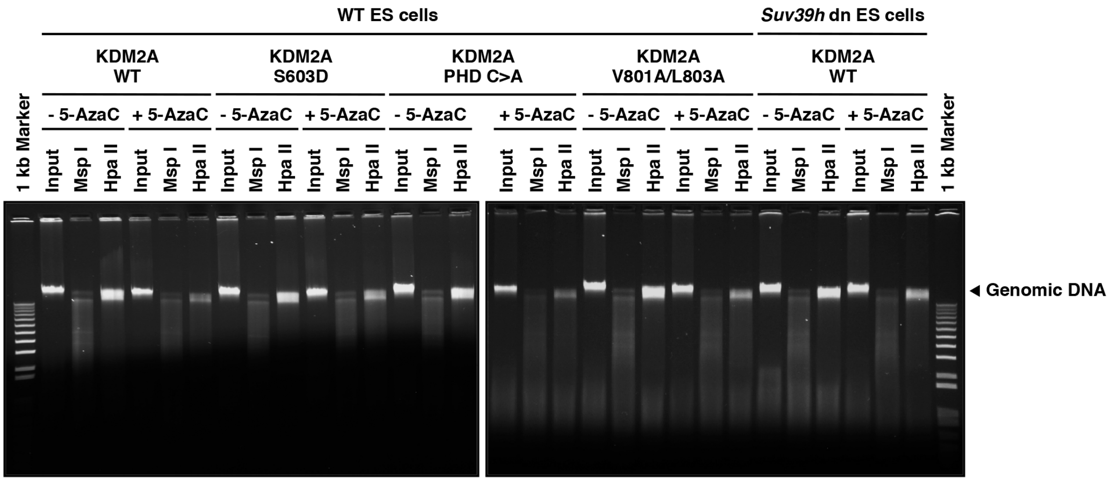

Supplementary Figure S6. The interaction between KDM2A and HP1 is required for targeting of KDM2A to heterochromatic repeats *in vivo*, related to Figure 5.

Effectiveness of the 5-Azacytidine treatment of the mouse ES cells used in Figure 5. Genomic DNA was prepared from WT or *Suv39h* dn mouse ES cells transfected with FLAG-GFP-KDM2A WT or mutant expression constructs and treated with 5-Azacytidine (5-AzaC) as indicated. For each sample 500 ng of genomic DNA were digested over night with 20 units of the restriction enzymes Hpa II or Msp I and 500 ng of the inputs and digests were separated on a 0.7% agarose gel. DNA was visualised by ethidium bromide staining. Hpa II cuts DNA at unmethylated CCGG sites and is blocked by genomic CpG methylation. In the untreated samples the Hpa II digest does not lead to a visible reduction of signal in the genomic DNA band indicating high DNA methylation levels. In all 5-Azacytidine treated samples digestion with Hpa II results in a substantial loss of signal for the genomic DNA indicating that the 5-Azacytidine treatment was effective in reducing overall genomic DNA methylation levels. Msp I cuts DNA at CCGG sites, similar to Hpa II, but is not methylation sensitive and completely digests the genomic DNA in all cases, serving as a control for the presence of the restriction sites.

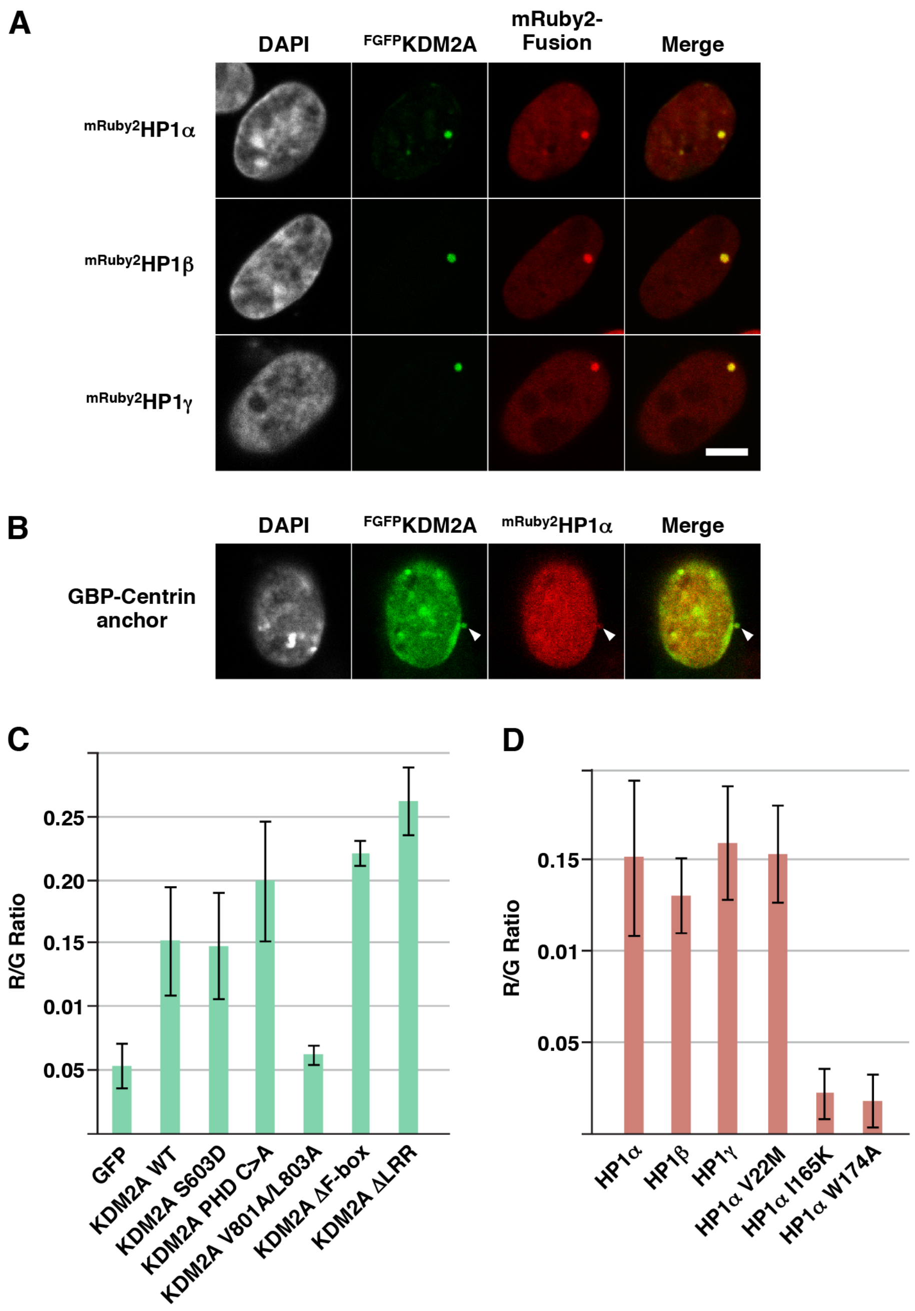

Supplementary Figure S7. KDM2A interacts with all HP1 isoforms *in vivo*, related to Figure 6.

(A) Cell-based F3H interaction assay between WT KDM2A and HP1 isoforms. The assay was carried out as described in Figure 6. mRuby2-tagged HP1α, HP1β and HP1γ co-localise with GFP-tagged KDM2A at the ectopic genomic

*lac*O array locus, showing an interaction between all HP1 isoforms and KDM2A. Scale bar represents for 5 μm. For quantification see Figure S7D.

(B) Tethering GFP-KDM2A to the centrosome in the cytoplasm using a GBP-centrin anchor recruits mRuby2-HP1α, indicating an interaction between the two proteins (arrowhead).

(C) Quantitative analysis of the binding assay carried out in Figure 6A illustrating the relative amount of mRuby2-HP1α recruited by different KDM2A point and deletion mutants. Relative intensity of mRuby2 to GFP fluorescence at the *lac*O spot are calculated for each KDM2A mutant, the KDM2A V801A/L803A mutant shows little to no interaction with HP1α.

(D) Quantitative analysis of the impact of different HP1α mutations on the interaction with KDM2A. The I165K and W174A mutation abolish the interaction between HP1α and KDM2A, but not the V22M mutation. For each sample 15 to 20 cells were measured, the standard deviation is shown as error bar.

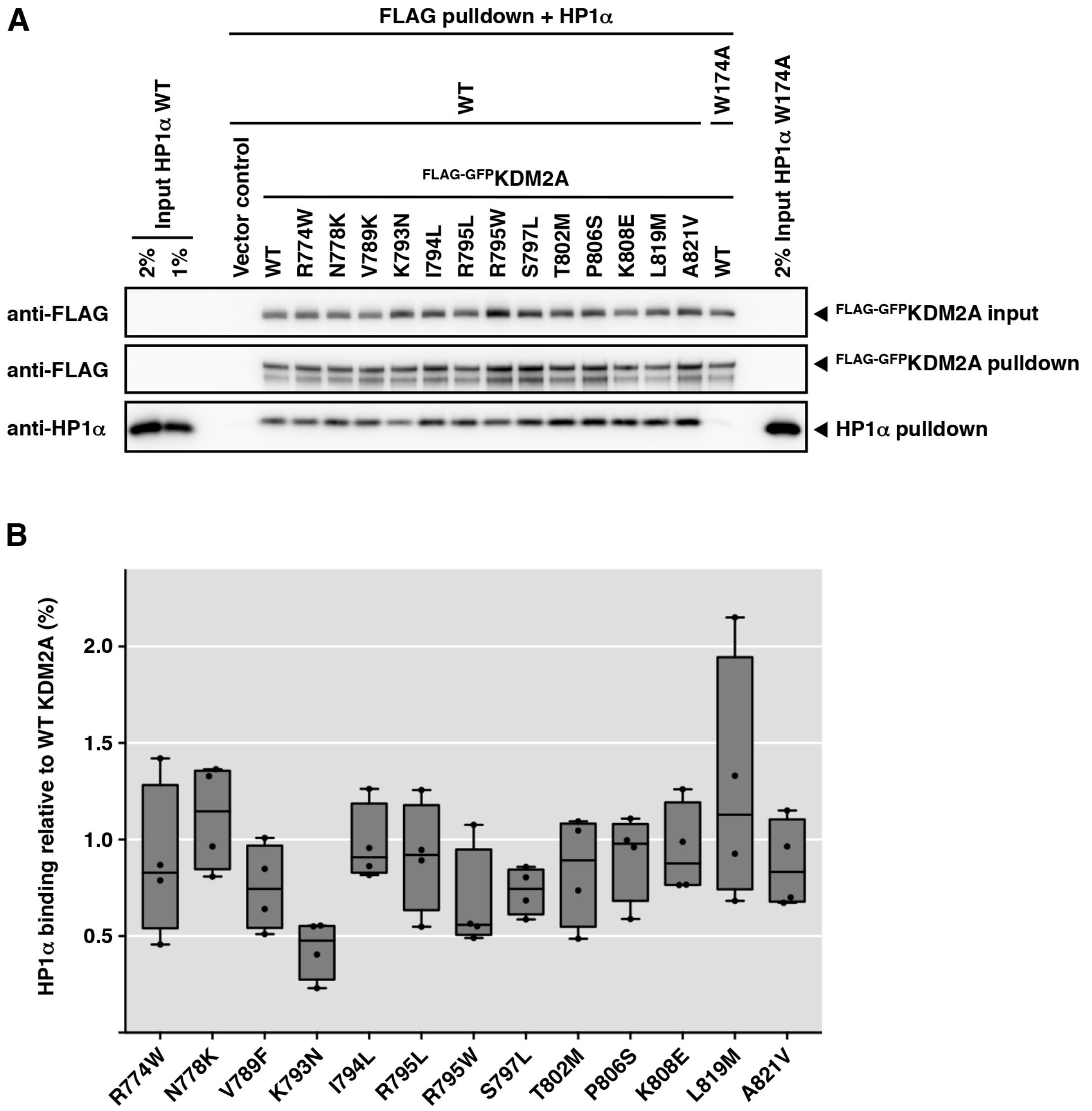

Supplementary Figure S8. KDM2A cancer mutations N-terminal of the HP1 binding motif impair the interaction between KDM2A and HP1α, related to Figure 7.

(A) Screen for KDM2A cancer mutations affecting the KDM2A/HP1 interaction. FLAG-GFP-tagged KDM2A WT or cancer mutants containing mutations surrounding the HP1 binding motif were expressed in 293T cells and captured on FLAG-affinity beads. The immobilised KDM2A proteins were then incubated with 2 μg of recombinant HP1α. 40% of the pull-down

reactions were resolved by SDS-PAGE and bound proteins detected by immunoblot against the FLAG tag (KDM2A) or HP1α using a chemiluminescence imaging system. The figure shows the immunoblots obtained for a representative pull-down experiment. Shown are anti-FLAG immunoblots for the KDM2A expression-adjusted 293T extracts (input) and for the FLAG-GFP-KDM2A present on the beads after the pull-down, and the anti-HP1α immunoblot to detect KDM2A-bound HP1α. Pull-downs with empty beads, and with WT KDM2A and the HP1α W174A mutant demonstrate the specificity of the interaction.

(B) Quantification of the immunoblots for the screen for KDM2A cancer mutants affecting the KDM2A/HP1α interaction shown in Figure S8A. The chemiluminescence signals associated with the full length FLAG-GFP-KDM2A and HP1α bands in the FLAG and HP1α immunoblots, respectively, were quantified for each pull-down sample. The HP1α signal intensities in the mutants were calculated relative to the signal in the WT KDM2A pull-down using the FLAG (KDM2A) signals obtained for the pull-downs with the respective mutants for normalisation. The figure shows the box plots and distributions from four independent experiments. The V789F, R795W and S797L mutants show potentially weak impairment of the KDM2A/HP1 interaction. The K793N mutation leads to a significant reduction of binding to ~50% of the WT.

A

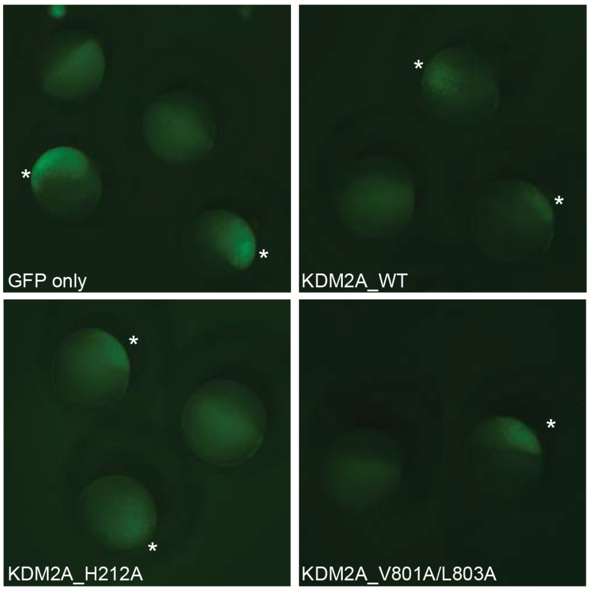

B

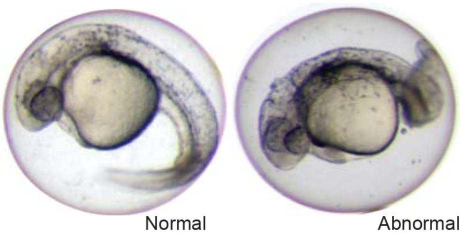

C

Expt #	Comparison	p-value	adj. p-value	Odds ratio
1	GFP-vs-KDM2A_V801A/L803A	0.347	1	0.720
2	GFP-vs-KDM2A_V801A/L803A	0.903	1	1.034
3	GFP-vs-KDM2A_V801A/L803A	0.022	0.065	1.681
4	GFP-vs-KDM2A_V801A/L803A	0.461	1	1.220
1	GFP-vs-KDM2A_WT	0.319	0.958	1.367
2	GFP-vs-KDM2A_WT	7.00E-07	**2.00E-06**	3.031
3	GFP-vs-KDM2A_WT	5.41.E-09	**1.62E-08**	3.576
4	GFP-vs-KDM2A_WT	0.004	**0.013**	1.996
1	KDM2A_V801A/L803A-vs-KDM2A_WT	0.024	0.071	1.900
2	KDM2A_V801A/L803A-vs-KDM2A_WT	1.60E-06	**4.80E-06**	2.933
3	KDM2A_V801A/L803A-vs-KDM2A_WT	3.93E-04	**0.001**	2.127
4	KDM2A_V801A/L803A-vs-KDM2A_WT	0.037	0.112	1.636
5	GFP-vs-KDM2A_H212A	**1.58E-04**	n/a	2.422
6	GFP-vs-KDM2A_H212A	**0.007**	n/a	2.014
7	GFP-vs-KDM2A_H212A	0.902	n/a	0.942
8	GFP-vs-KDM2A_H212A	**7.00E-07**	n/a	4.095

D

# eggs scored	1	2	3	4	5	6	7	8
GFP	100	180	181	149	180	180	180	180
KDM2A_V801A/L803A	150	168	185	160				
KDM2A_H212A					147	161	180	153
KDM2A_WT	150	186	184	154				

E

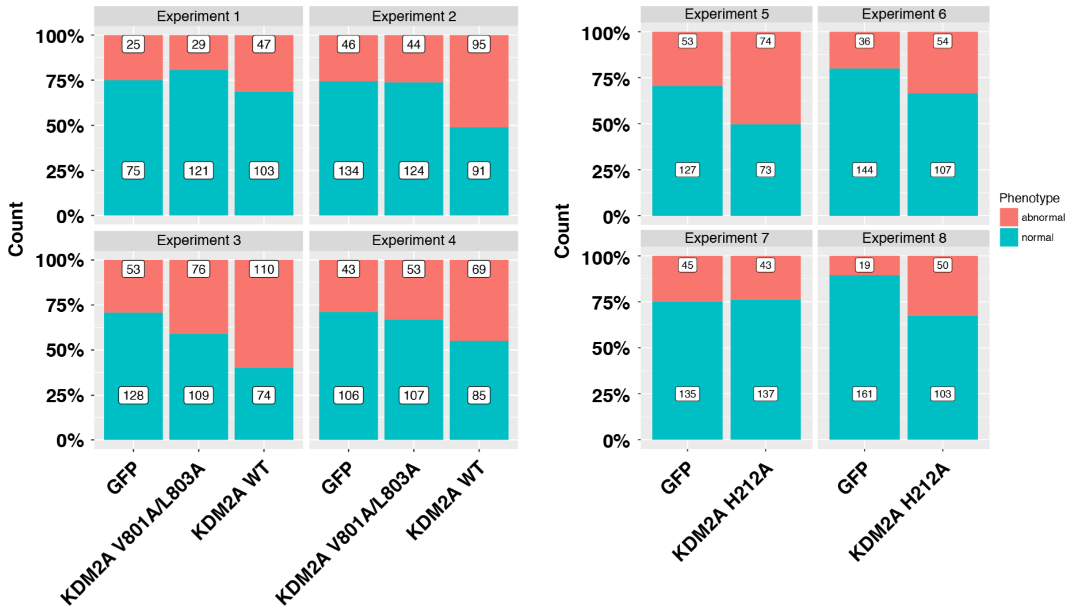

Supplementary Figure S9. Expression of FLAG-GFP-tagged KDM2A constructs in zebrafish embryos and overexpression phenotype, related to Figure 7.

(A) GFP expression at dome stage in zebrafish eggs injected with 3.6 nl of 250ng/µl mRNA synthesised from constructs containing either FLAG-GFP

alone or versions of FLAG-GFP-tagged human KDM2A. Expressing embryos are denoted by an asterisk. Non-expressing embryos only display weak auto-fluorescence of the yolk.

(B) Examples of embryos at 24 h.p.f. showing normal and abnormal phenotypes.

(C) Table of individual overexpression experiments shown in Figure 7D with Fisher's exact test p-value, adjusted p-value (where appropriate) and odds ratio.

(D) Table of number of eggs scored per injection for overexpression experiments shown in Figure 7D.

(E) Bar charts of the counts for normal and abnormal embryos in each of the individual overexpression experiments shown in Figure 7D.

SUPPLEMENTARY MATERIAL AND METHODS

Plasmids

The constructs for the bacterial expression of human core histone proteins and for the tail-less H3.1Δ1-31T32C protein for native chemical ligations were described previously (3). pUC19 vector containing 16 tandem repeats of the 601 nucleosome-positioning sequence flanked by EcoRV and EcoRI sites was described previously (3). The pGBP-LacI vector for mammalian expression of LacI-fused GFP binder protein was described previously (4). The expression constructs for mammalian expression of FLAG-tagged and mRuby2-tagged human HP1 proteins were generated by RT-PCR from HeLa S3 total RNA (HP1α) or obtained by gene synthesis from Genscript (HP1β and HP1γ) and cloned into pcDNA5/FRT/TO-based expression vectors. The bacterial GST-HP1α, GST-HP1β, and GST-HP1γ expression constructs (murine HP1 proteins) were a kind gift from Tony Kouzarides. Constructs for FLAG-GFP-tagged full-length KDM2A and N- and C-terminal deletion mutants were generated by PCR from a FLAG-KDM2A construct kindly provided by Yi Zhang (5) and cloned into a pcDNA5/FRT/TO-based expression vector for transfection into mammalian cells and for generating mRNA for injections into zebrafish embryos. The bacterial expression construct for His_6-SUMO-$KDM2A_{543\text{-}811}$ was generated by PCR from the FLAG-KDM2A construct and cloned into pCA528 (kind gift from Bernd Bukau) (6). Point mutations were introduced by site-directed mutagenesis (QuickChange II XL, Agilent Technologies). All constructs were verified by sequencing.

Antibodies

Antibodies for immunoblots directed against histone H3K4me3 (ab8580) and H3K9me3 (ab8898) were rabbit polyclonals obtained from Abcam. Anti-HP1α antibodies for immunoblots were either polyclonal rabbit from Cell Signaling (2616), monoclonal rabbit from Abcam (ab109028), polyclonal goat from Abcam (ab77256), or a mouse monoclonal from Merck/Millipore (MAB3446). The mouse monoclonal anti-HP1α antibody used for

immunofluorescence was from Merck/Millipore (MAB3584). Mouse monoclonal anti-HP1β (MAB4338) and anti HP1γ (MAB 3450) antibodies were from Merck/Millipore. Anti-Flag antibodies were either the M2 monoclonal from Sigma or a goat polyclonal from Abcam (ab1257). The polyclonal rabbit anti-GFP antibodies used for immunoprecipitation (A-11122) and immunofluorescence (Alexa Fluor® 488 conjugate; A-21311) were from LifeTechnologies. Rabbit anti-KDM2A antibody used for immunoprecipitation and immunoblots was from Bethyl Laboratories (A301-475A). A new rabbit anti-KDM2A antibody that recognises all potential isoforms of KDM2A was raised and affinity purified against a recombinantly expressed fragment of KDM2A spanning amino acids 689 to 779 and used for immunoblots.

Protein Expression and Purification

Recombinant histone proteins were expressed in *E. coli* BL21(DE3)/RIL cells from pET21b(+) (Novagen) vectors and purified by denaturing gel filtration and ion exchange chromatography essentially as described (7). The tail-less H3.1Δ1-31T32C protein for native chemical ligations was purified as described (3). All histone proteins were stored lyophilised at -80°C. Recombinant HP1α and its point mutants were expressed in *E. coli* BL21(DE3)/RIL cells as GST-fusion proteins and purified by glutathione sepharose (GE Healthcare) chromatography. HP1α was cleaved off the beads with biotinylated thrombin (Novagen). After removal of thrombin with streptavidin sepharose (GE Healthcare) HP1α was dialysed into TBS/10% glycerol, snap frozen and stored at -80°C. Full length KDM2A was purified from 293T cells transiently transfected with pcDNA5/FRT/TO-based FLAG-GFP fusion constructs using a PEI protocol. Cleared lysates were prepared ~36 h after transfection by rotating the cells in extraction buffer (20 mM Hepes pH7.5; 300 mM NaCl; 1 mM EDTA; 20% Glycerol; 0.5% NP40; 1 mM PMSF and complete protease inhibitors) for 1 h at 4°C and removing cell debris by centrifugation. FLAG-GFP-KDM2A was captured on anti-FLAG M2 agarose beads (Sigma) for 4 h at 4 °C, extensively washed with extraction buffer followed by wash buffer (20 mM Hepes pH7.5; 150 mM NaCl; 1 mM EDTA;

20% Glycerol; 0.1% NP40; 1 mM PMSF and complete protease inhibitors), and eluted in wash buffer without NP40 containing 0.25 mg/ml 3xFLAG peptide (Sigma). Eluted proteins were directly aliquoted and snap frozen. Concentrations were determined by SDS-PAGE and Coomassie stain against a BSA standard. $KDM2A_{543-811}$ was expressed from pCA528 as an N-terminal His_6-SUMO fusion in BL21(DE3)/RIL cells grown in 2YT media containing 0.1 mM $ZnCl_2$. Cells were sonicated in lysis buffer (50 mM HEPES-KOH pH 7.4, 500 mM NaCl, 5% Glycerol, 5 mM Imidazol, 0.5 mM TCEP) containing complete protease inhibitors. The soluble fraction was purified on a Ni-Sepharose affinity column (GE Healthcare) using a standard protocol. The His-SUMO tag was cleaved off with His_6-Ulp1 sumo protease during an overnight dialysis step into 50 mM Tris pH 7.5, 500 mM NaCl, 10% Glycerol, 2 mM beta-ME. The protein was further purified on a Superdex S75 gel filtration column (GE Healthcare) in gel filtration buffer (20 mM Tris pH 7.5, 150 mM NaCl, 5% Glycerol) followed by a Heparin column (GE Healthcare) equilibrated in buffer A (50 mM Tris pH 7.5, 150 mM NaCl, 5% Glycerol). Proteins were eluted over a 10 CV gradient of 0-100% buffer B (50 mM Tris pH 7.5, 2 M NaCl, 5% Glycerol). $KDM2A_{543-811}$ fractions were pooled, snap frozen and stored at -80°C or further purified by an optional Superdex S75 gel filtration step as above.

SUPPLEMENTARY FILES

Supplementary File 1. KDM2A Sequence Alignments, related to Figure 7.

Sequence alignment of the full length protein sequences of KDM2A and its orthologues in different species representing different levels of phylogenetic depth. The KDM2A sequences are aligned with human KDM2B and human Fbxl19 for comparison.

SUPPLEMENTARY TABLES

Supplementary Table 1: SNPs and cancer SNVs identified in KDM2A, related to Figure 7.

The file contains a list of missense SNPs and cancer SNVs identified in KDM2A including the chromosomal location of the mutation, the nucleotide change and the resulting amino acid change in the protein.

SUPPLEMENTARY REFERENCES

1. Lechner, M.S., Schultz, D.C., Negorev, D., Maul, G.G. and Rauscher, F.J., 3rd. (2005) The mammalian heterochromatin protein 1 binds diverse nuclear proteins through a common motif that targets the chromoshadow domain. *Biochemical and biophysical research communications*, **331**, 929-937.

2. Brasher, S.V., Smith, B.O., Fogh, R.H., Nietlispach, D., Thiru, A., Nielsen, P.R., Broadhurst, R.W., Ball, L.J., Murzina, N.V. and Laue, E.D. (2000) The structure of mouse HP1 suggests a unique mode of single peptide recognition by the shadow chromo domain dimer. *The EMBO journal*, **19**, 1587-1597.

3. Bartke, T., Vermeulen, M., Xhemalce, B., Robson, S.C., Mann, M. and Kouzarides, T. (2010) Nucleosome-interacting proteins regulated by DNA and histone methylation. *Cell*, **143**, 470-484.

4. Herce, H.D., Deng, W., Helma, J., Leonhardt, H. and Cardoso, M.C. (2013) Visualization and targeted disruption of protein interactions in living cells. *Nature communications*, **4**, 2660.

5. Tsukada, Y., Fang, J., Erdjument-Bromage, H., Warren, M.E., Borchers, C.H., Tempst, P. and Zhang, Y. (2006) Histone demethylation by a family of JmjC domain-containing proteins. *Nature*, **439**, 811-816.

6. Andreasson, C., Fiaux, J., Rampelt, H., Mayer, M.P. and Bukau, B. (2008) Hsp110 is a nucleotide-activated exchange factor for Hsp70. *The Journal of biological chemistry*, **283**, 8877-8884.

7. Dyer, P.N., Edayathumangalam, R.S., White, C.L., Bao, Y., Chakravarthy, S., Muthurajan, U.M. and Luger, K. (2004) Reconstitution of nucleosome core particles from recombinant histones and DNA. *Methods in enzymology*, **375**, 23-44.
