# Supplementary figures and images for: KDM2A integrates DNA and histone modification signals through a CXXC/PHD module and direct interaction with HP1
Source: Nucleic Acids Res. 2016 Oct 24;45(3):1114–29. doi: 10.1093/nar/gkw979 (PMC5388433; doi:10.1093/nar/gkw979)

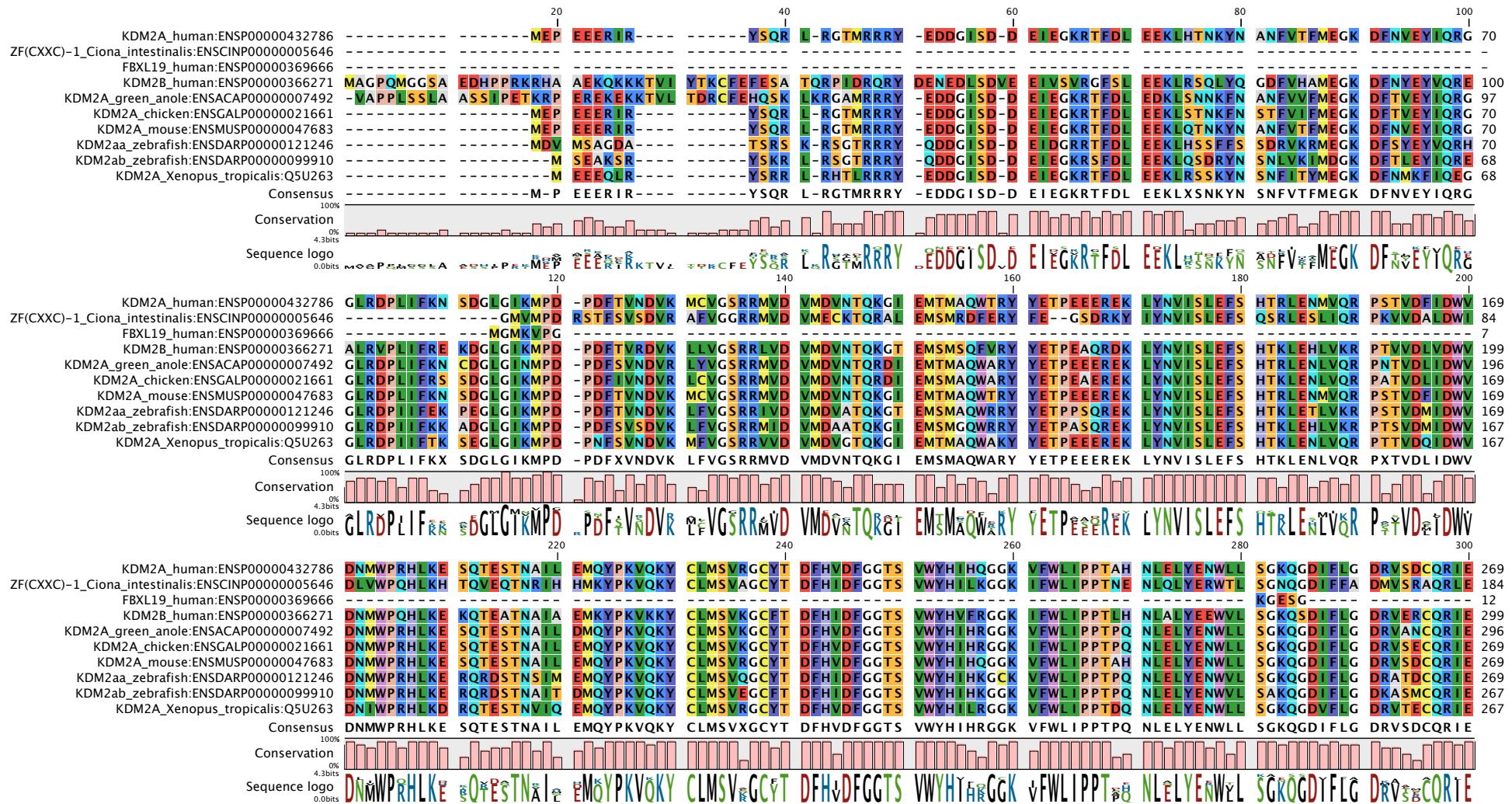

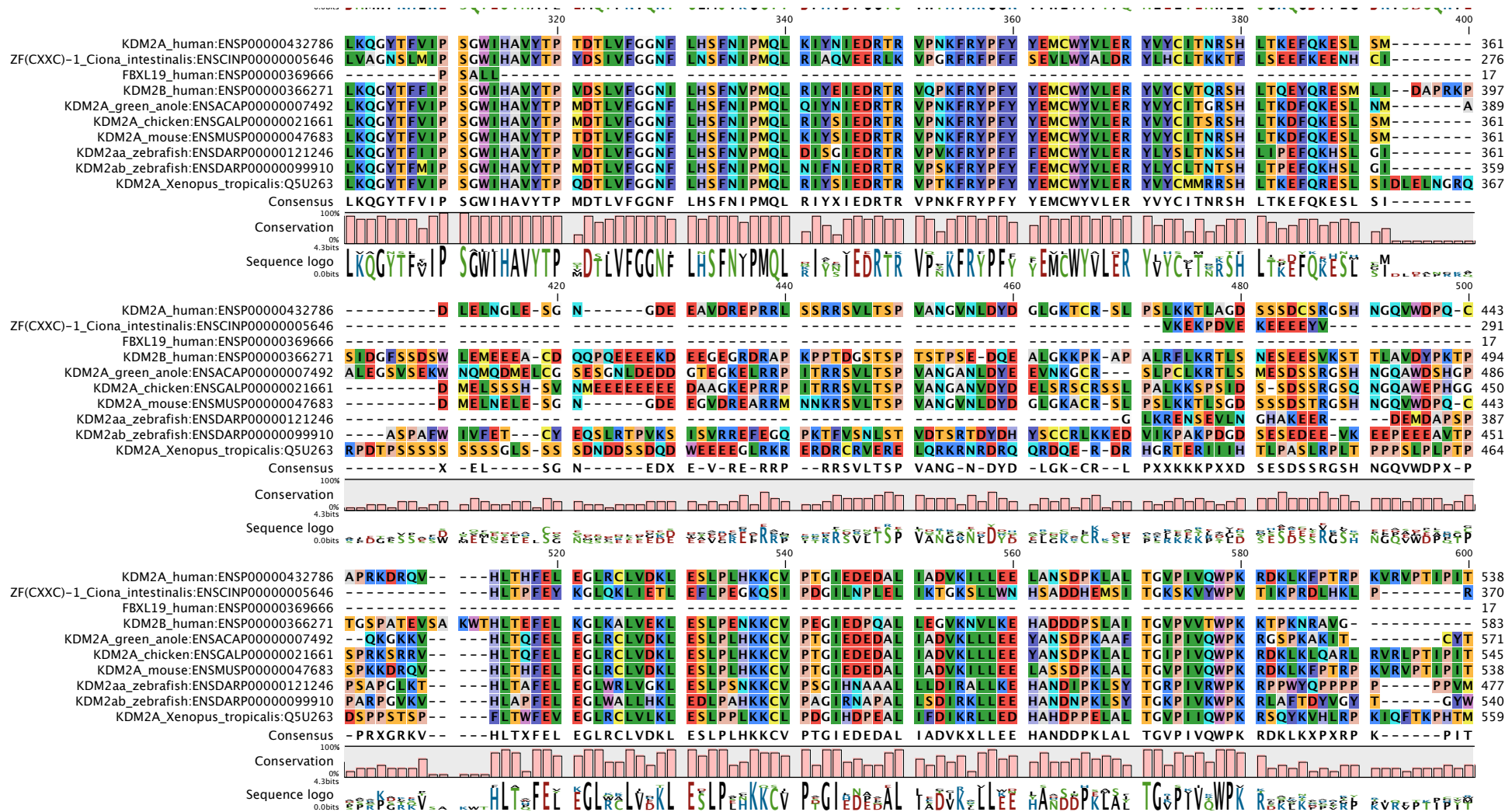

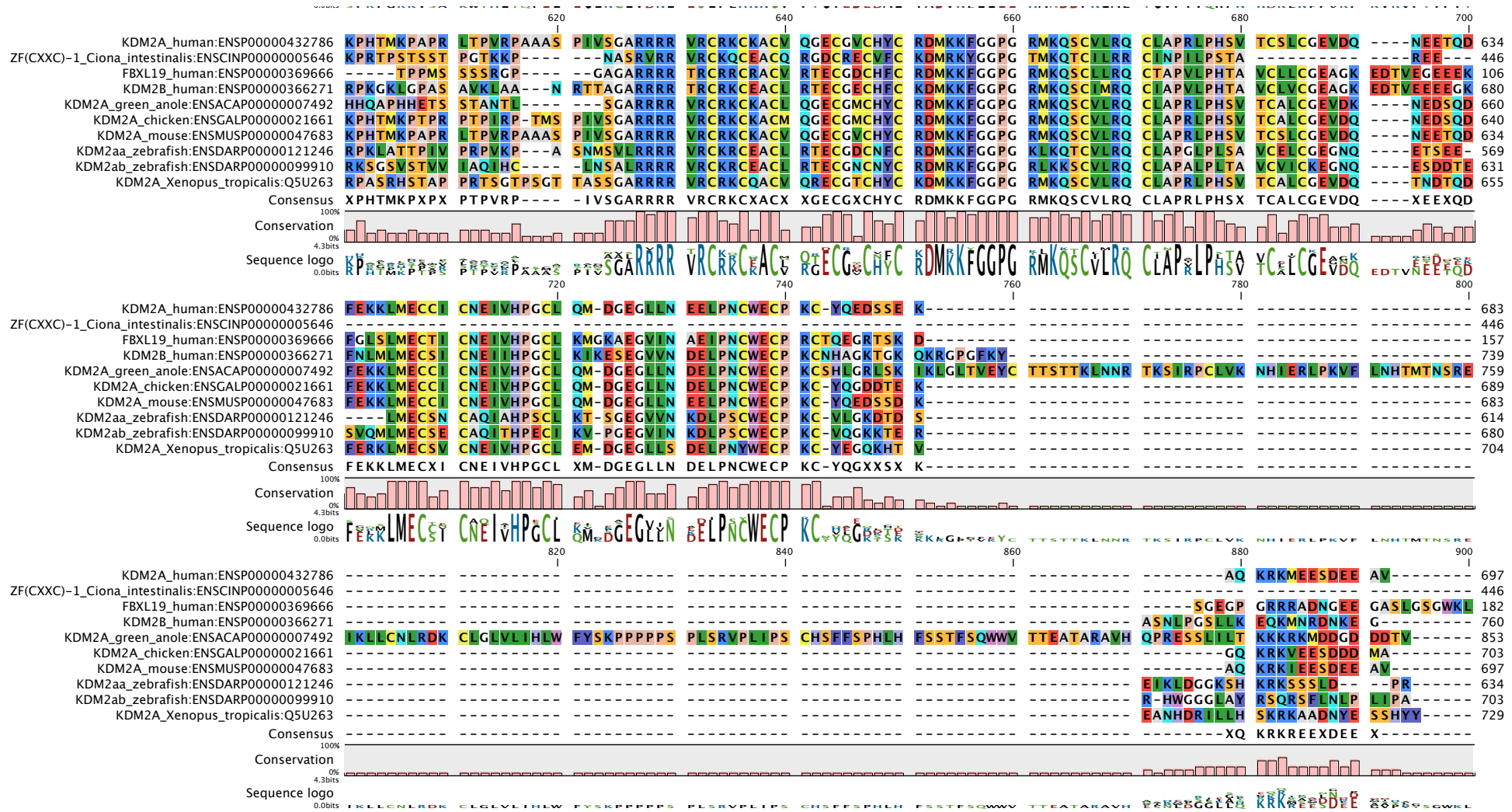

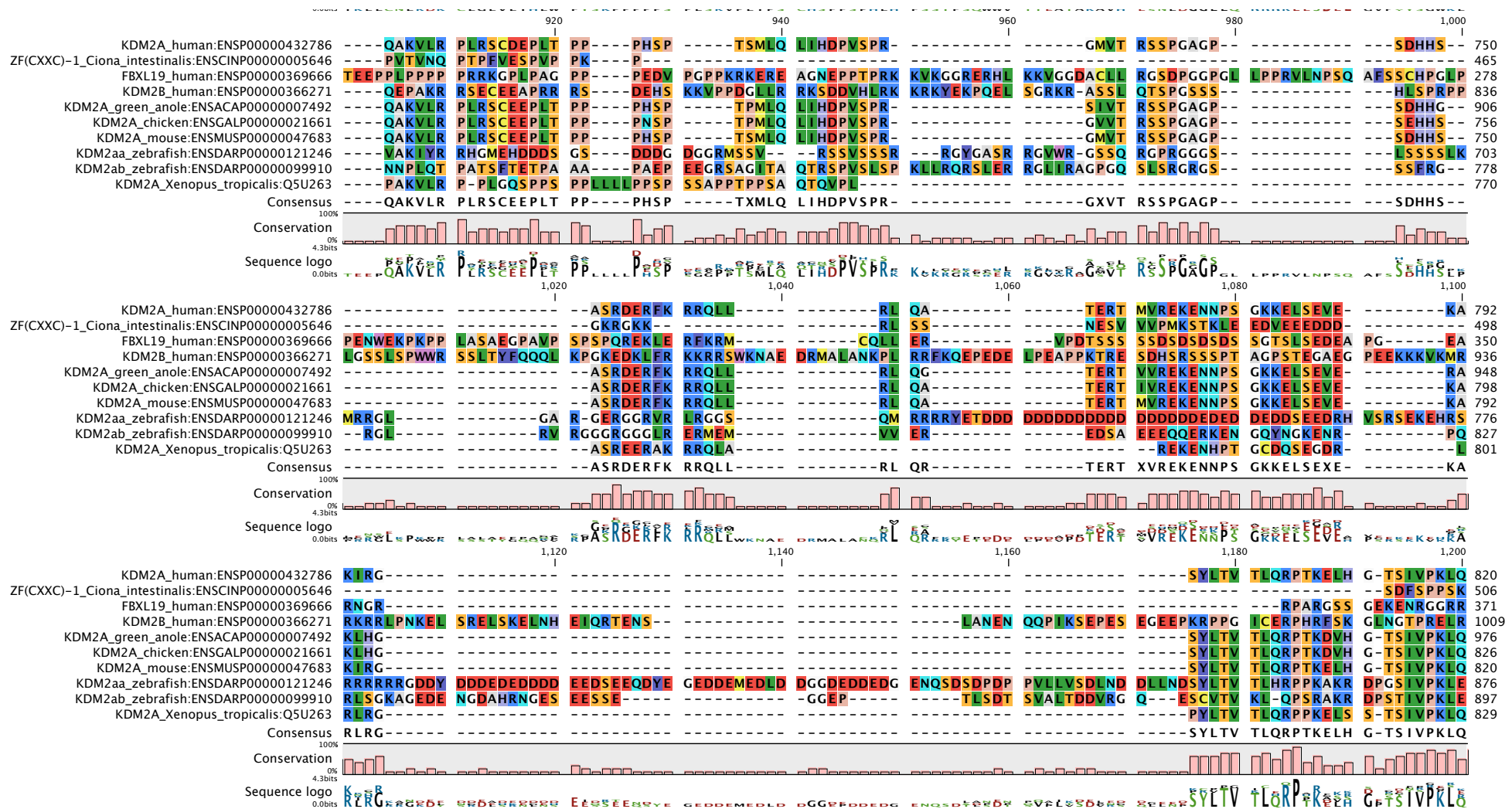

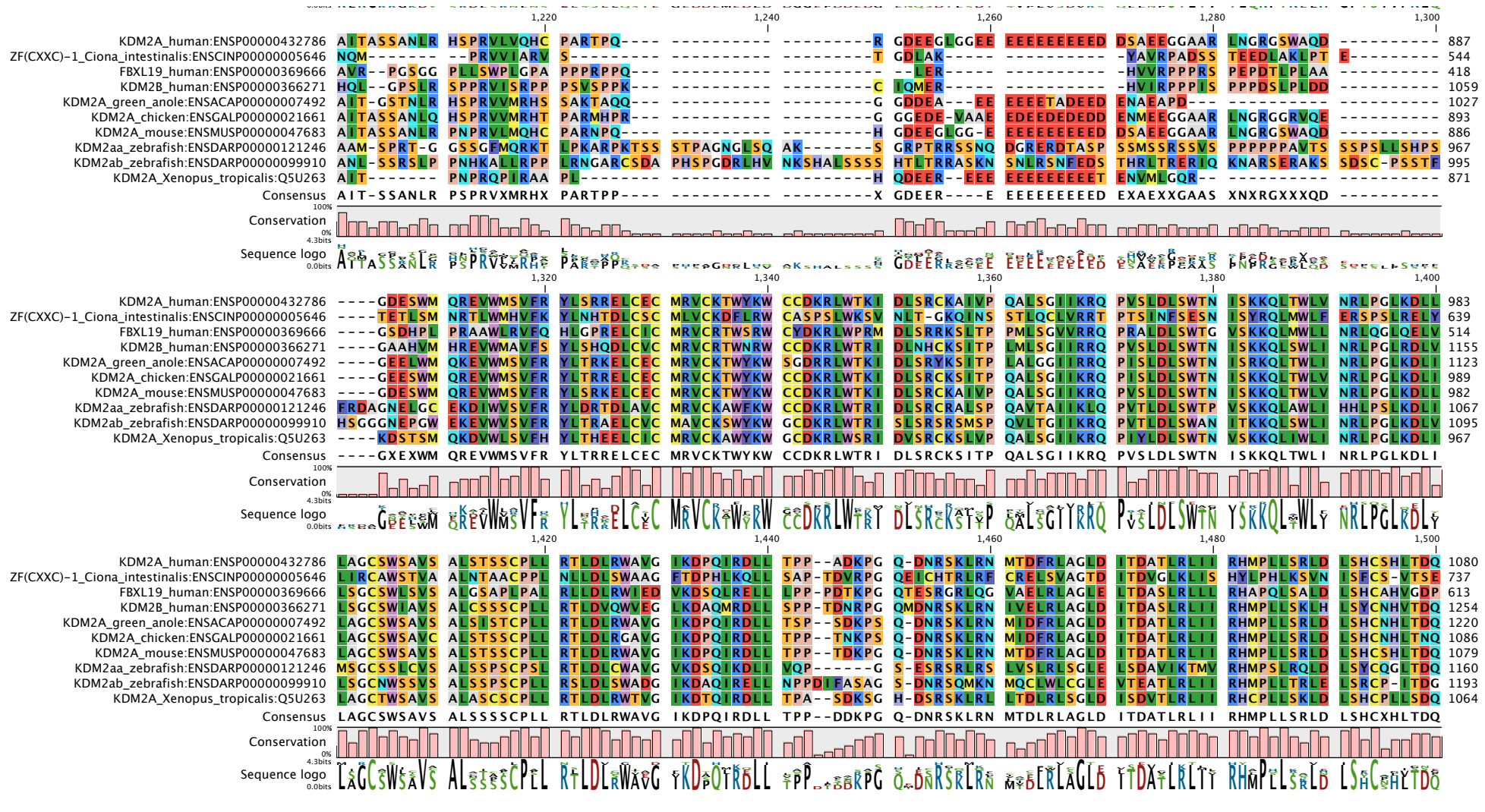

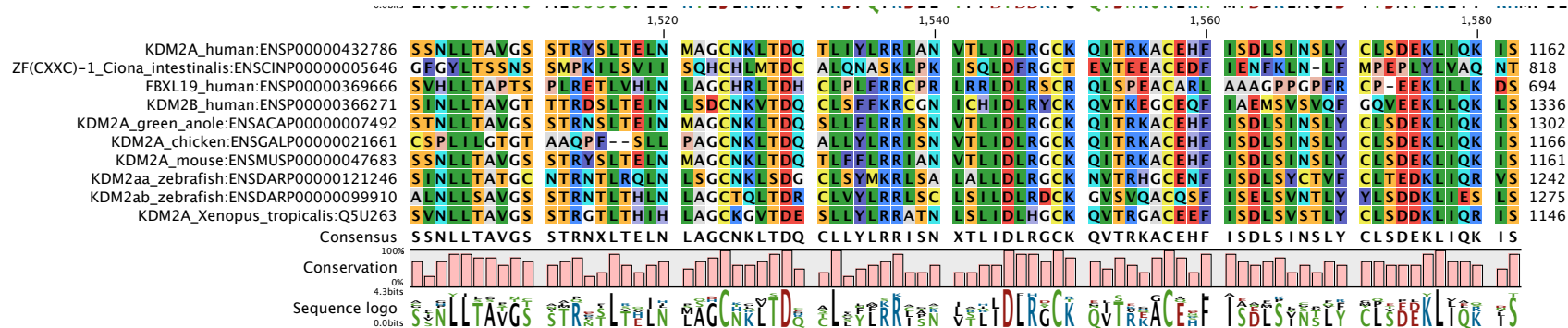

Supplement: Supplementary Data [file gkw979_Supp.zip › nar-00658-m-2016-File011.pdf]
